# Discovery of potent inhibitors of α-synuclein aggregation using structure-based iterative learning

**DOI:** 10.1038/s41589-024-01580-x

**Published:** 2024-04-17

**Authors:** Robert I. Horne, Ewa A. Andrzejewska, Parvez Alam, Z. Faidon Brotzakis, Ankit Srivastava, Alice Aubert, Magdalena Nowinska, Rebecca C. Gregory, Roxine Staats, Andrea Possenti, Sean Chia, Pietro Sormanni, Bernardino Ghetti, Byron Caughey, Tuomas P. J. Knowles, Michele Vendruscolo

**Affiliations:** 1 Centre for Misfolding Diseases, Yusuf Hamied Department of Chemistry, University of Cambridge, Cambridge, UK; 2 Laboratory of Neurological Infections and Immunity, Rocky Mountain Laboratories, National Institute for Allergy and Infectious Diseases, National Institutes of Health, Hamilton, MT USA; 3 Bioprocessing Technology Institute, Agency for Science, Technology and Research (A*STAR), Singapore, Singapore; 4 Department of Pathology and Laboratory Medicine, Indiana University School of Medicine, Indianapolis, IN USA

**Keywords:** Drug discovery, Computational biology and bioinformatics, Single-molecule biophysics, Neurodegenerative diseases, Cheminformatics

## Abstract

Machine learning methods hold the promise to reduce the costs and the failure rates of conventional drug discovery pipelines. This issue is especially pressing for neurodegenerative diseases, where the development of disease-modifying drugs has been particularly challenging. To address this problem, we describe here a machine learning approach to identify small molecule inhibitors of α-synuclein aggregation, a process implicated in Parkinson’s disease and other synucleinopathies. Because the proliferation of α-synuclein aggregates takes place through autocatalytic secondary nucleation, we aim to identify compounds that bind the catalytic sites on the surface of the aggregates. To achieve this goal, we use structure-based machine learning in an iterative manner to first identify and then progressively optimize secondary nucleation inhibitors. Our results demonstrate that this approach leads to the facile identification of compounds two orders of magnitude more potent than previously reported ones.

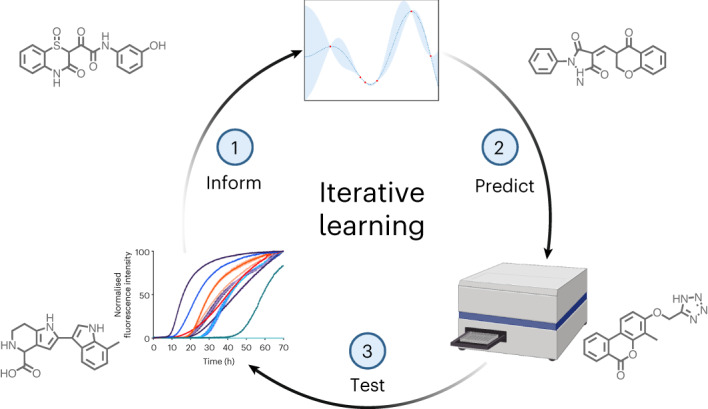

## Abstract (web summary)

Developing disease-modifying drugs for neurodegenerative diseases has been very challenging. Now a machine learning approach has been used to identify small molecule inhibitors of α-synuclein aggregation, a process implicated in Parkinson’s disease and other synucleinopathies. Compounds that bind to the catalytic sites on the surface of the aggregates were identified and then progressively optimized into secondary nucleation inhibitors.

## Main

Parkinson’s disease (PD) is the most common neurodegenerative movement disorder, affecting 2–3% of the population over 65 years of age^1–5^. The aggregation of α-synuclein (αS) has been associated with the initial neurodegenerative processes underlying this disease, in which the pathological accumulation of misfolded proteins results in neuronal toxicity. Motor symptoms appear once this pathology affects the substantia nigra^1,2,4,6^. Since αS aggregates have been shown to exhibit various mechanisms of cellular toxicity^7,8^, major efforts are being invested into identifying compounds that can inhibit αS aggregation mechanisms^9–12^. This is a particularly pressing need given the lack of disease-modifying therapies currently available to patients with PD^13–15^. With the recent approval by the US Food and Drug Administration of the first two disease-modifying drugs for Alzheimer’s disease, aducanumab^16^ and lecanemab^17^, approaches based on blocking secondary nucleation appear to be promising^18^.

Computational methods could be expected to reduce the time and cost of traditional drug discovery pipelines^19–21^. In this area, machine learning is rapidly emerging as a powerful drug discovery strategy^22^. In this Article, to explore the potential of this strategy in drug discovery programs for PD and other synucleinopathies, we describe a machine learning approach to explore the chemical space to identify compounds that inhibit the aggregation of αS. Our starting point is an approach that combines docking simulations with in vitro screening, which was recently employed to identify a set of compounds that bind to the fibril structures of αS, and prevent the autocatalytic proliferation of αS fibrils as a result^23^. Here we used this initial set of compounds as input for a structure-based machine learning approach to identify chemical matter that is both efficacious and represents a substantial departure from the parent structures. This provided compounds that conventional similarity searches would have failed to efficiently identify.

This approach is based on the lessons learned using chemical kinetics about the importance of secondary nucleation in αS aggregation^24–26^. Because of the autocatalytic nature of this process, structure-based methods could be expected to effectively target the catalytic sites on the surface of αS aggregates^23^. As we show here, the implementation of this idea within an iterative machine learning procedure leads to the identification and optimization of compounds with great potency.

## Results

### Components of the machine learning method

The machine learning approach used here consists of three main components^27^: (1) the experimental data, which is a readout of the potency of the compounds in an aggregation assay, (2) the variational autoencoder required to represent the compounds as latent vectors, and (3) a model for training and prediction using these vectors and the assay readouts.

For component 1, we used a chemical kinetics assay^9,28,29^ that provided both the initial data for the model training and the data that were iteratively fed back into the model at each cycle of testing and prediction. This assay identifies the top compounds that inhibit the surface-catalyzed secondary nucleation step in the aggregation of αS. Secondary nucleation is enabled by adding a small amount of preformed fibrils to a monomeric mixture. Aggregation was tracked using the amyloid binding dye, thioflavin T (ThT).

For component 2, we used a junction tree variational autoencoder^30^, pretrained on a set of 250,000 molecules^31^ enabling accurate representation of a diverse population of molecular structures. Using this approach, SMILES strings were standardized using MolVS^32^ and converted into latent vector representations.

**Fig. 1:**
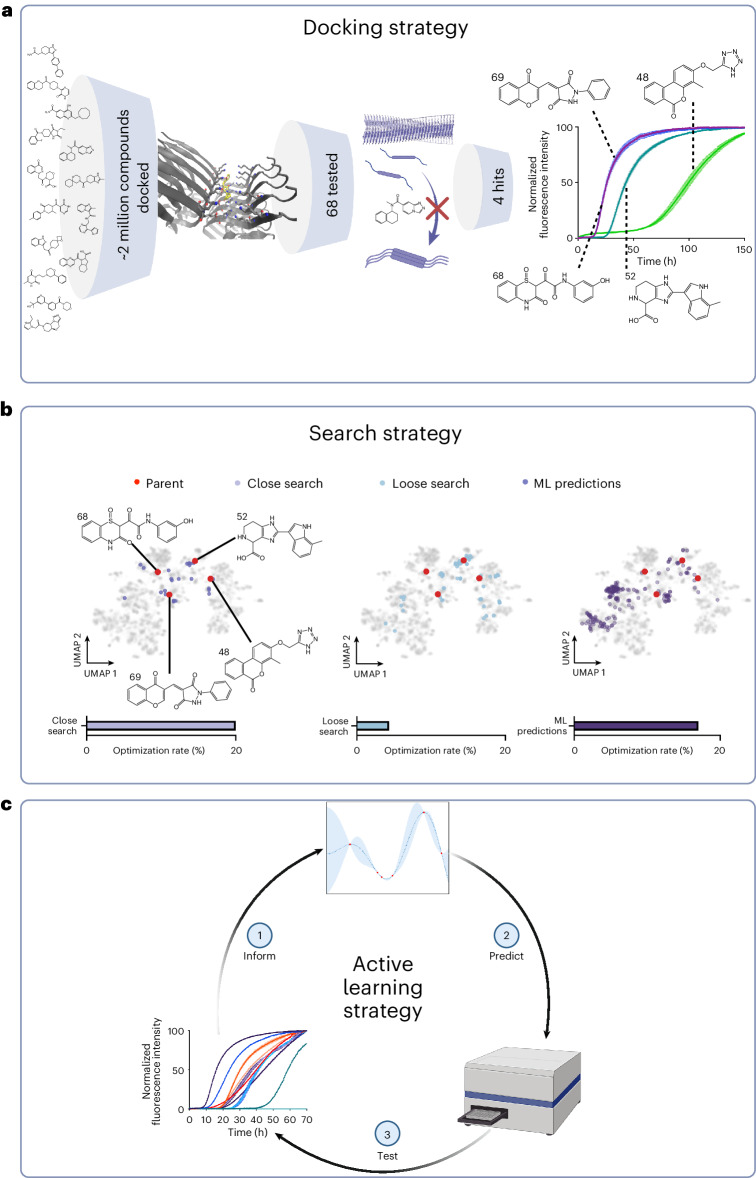
Illustration of the three stages of exploration of the chemical space described in this work. **a**, From 68 molecules predicted to have good binding via docking simulations, we initially identified 4 active molecules (the ‘docking set’) by experimental testing^23^. These four molecules increase the *t*_1/2_ of αS aggregation. **b**, We then performed a close Tanimoto similarity search around the four parent compounds in chemical space. We selected molecules with Tanimoto similarity cutoff >0.5 (the ‘close similarity docking set’) followed by a loose similarity search with Tanimoto similarity cutoff >0.4 (the ‘loose similarity docking set’). A machine learning method was then applied using the observed data to predict potent molecules from a compound library derived from the ZINC database with Tanimoto similarity >0.3 to the parent structures (the ‘evaluation set’). **c**, Successive iterations of prediction and experimental testing yielded higher optimization rates (defined as the percentage of molecules increasing the normalized half time of aggregation above 2), and molecules with higher potency on average than those identified in the previous similarity searches. Validation experiments were also carried out on the potent molecules identified.

For component 3, we used a random forest regressor (RFR) with a Gaussian process regressor (GPR) fitted to the residuals^33,34^ of the RFR, with both regressors using the latent vectors as training features. The RFR provided the highest performance compared to other combinations of multilayer perceptrons (MLPs), GPRs and linear regressors (LRs) in terms of *R*^2^ score, mean absolute error and root mean square error. Performance and parameters are shown in Supplementary Fig. 1 and Supplementary Table 1, respectively. Combining the RFR and GPR provided only a marginal improvement in the metrics of the RFR alone, but crucially enabled leveraging of the associated uncertainty measure of the GPR when ranking molecules during acquirement prioritization^27^. Tuning the weighting applied to this uncertainty measure allowed a ranking based on both the predicted potency of the molecules and the uncertainty of that prediction. Component 3 was then trained on the 161 initial experimental data points (see below). The best molecules predicted by the model were then tested in the same assay and the results fed back into the model in an iterative fashion (~55–65 new molecules tested at each iteration). The molecules used at each stage of the project are illustrated in Supplementary Fig. 2, together with the structures of the most potent hits and leads at each stage. An overview of the pipeline is shown in Fig. 1.

### Initial set of small molecules

The initial set of molecules was identified via docking simulations to αS fibrils (Supplementary Information), followed by similarity searches around molecules that performed well in the chemical kinetics assay to identify further candidates^23^. The docking screening was carried out using the consensus strong binders predicted by AutoDock Vina^35^ and Openeye’s FRED^36–38^ software.

Two million molecules with optimal central nervous system multiparameter optimization (CNS MPO)^39^ properties were previously docked using AutoDock Vina to target the selected binding pocket^23^ (Supplementary Fig. 3). CNS MPO is an aggregated metric of molecular properties that predicts likelihood of a molecule passing the blood–brain barrier. In that study, the binding site encompassing residues His50–Lys58 and Thr72–Val77 was selected due to its propensity to form a pocket according to the Fpocket software^37^ (Supplementary Fig. 3a), and its mid to low solubility according to CamSol^40^ (Supplementary Fig. 3b). Additionally, His50 is predicted to be protonated below the pH value (5.8) at which αS secondary nucleation more readily occurs^41^, which may be important for initial interactions. To increase the confidence of the calculations, the top-scoring 100,000 small molecules were selected and docked against the same αS binding site, using FRED^36^. The top-scoring, common 10,000 compounds in both docking protocols were selected and clustered using Tanimoto clustering^42^ with a similarity cutoff of 0.75, leading to a list of 79 centroids (representative molecules from each cluster). The Tanimoto similarity is a metric that compares Morgan fingerprint^43^ representations (radius 2, nbits 2,048) of two different molecules. A value of 1 for the Tanimoto similarity implies complete two-dimensional homology between two structures, while values closer to 0 imply little to no structural similarity. Sixty-eight compounds were available of the 79 molecules identified in the in silico structure-based docking study. The first round of in vitro experiments was carried out with this set.

Subsequent experiments to test these predicted binders in aggregation assays identified four active compounds^23^ labeled molecule 48, 52, 68 and 69, referred to as the ‘docking set’, (Fig. 1a). We then began the process of lead generation and optimization. Here, using the Tanimoto similarity metric between Morgan Fingerprint representations (radius 2, nbits 2,048) of the molecules, two similarity searches were then carried out on the ZINC15 database using these four structures as starting points (Fig. 1b). Different Tanimoto similarity thresholds were used to specify molecule subsets for testing. As such a similarity value >0.5 was used for closely related analogs, >0.4 for loosely related analogs and >0.3 for the library to screen from (‘evaluation set’). While this use of a structurally related screening library constrains the model’s ability to generalize, the lack of diversity in terms of potent molecules in the training set also makes it unlikely for the model to perform well in chemical space divergent from this region. We are thus carrying out an exploitation strategy here. We remove the need for a curated screening library in a parallel work by utilizing generative modeling and reinforcement learning^44^, allowing for both exploitation and exploration strategies.

A selection of closely related molecules (Tanimoto similarity >0.5) to the parent compounds (referred to as the ‘close similarity docking set’, Fig. 1b and Supplementary Fig. 2b) was tested in the aggregation assay. The potent molecule selection was made according to a cutoff corresponding to a normalized half-time of the aggregation (*t*_1/2_) of two times that of the negative control. The percentage of molecules passing this threshold was defined as the optimization rate. This yielded five new potent molecules from 25 new molecules (Supplementary Fig. 2b), 1 derived from molecule 48, three from molecule 52 and one from molecule 69. This step was then followed by a larger selection of compounds with a looser cutoff of structural similarity (Tanimoto similarity >0.4) to the parent compounds (referred to as the ‘loose similarity docking set’, Fig. 1b). Although new potent molecules featured among this set, the optimization rate was low (4%), and both molecules 48 and 52, which had initially appeared the most promising of the parent structures, yielded poor results. From the 29 molecules related to molecule 48 in the loose similarity docking set, none were potent, while from the 24 molecules related to molecule 52, only 2 were potent. The functional range of molecules 48 and 52 appeared narrowly limited around the chemical space of the parent structures. Molecule 69 yielded one potent molecule from 16 molecules. Overall, the optimization rate from the loose similarity docking set was less than a quarter of that of the close similarity docking set and involved testing three times as many compounds.

These results suggested that it would be challenging to further explore the chemical space using conventional structure–activity relationship techniques without considerable attrition, since the optimization rate worsened as the similarity constraint to the initial hits was loosened. To overcome this problem, the compounds resulting from these experiments were then used as input for a machine learning method for an iterative exploration of the chemical space (Fig. 1c). The similarity searches removed the most obvious targets of the machine learning approach, but also increased the size of the dataset available for training. The training set, however, remained small by typical machine learning standards, consisting of 161 molecules. Since training sets of this size are common in early-stage research, a further aim of this work was to demonstrate that machine learning can be used effectively even in such data-sparse scenarios.

### Iterative application of the machine learning approach

One of the issues with applying machine learning to a data-sparse scenario is that predictions are likely to be overconfident. While this problem can be addressed to an extent by utilizing Gaussian processes, a complementary strategy is to restrict the search area to a region of chemical space that is more likely to yield successful results. To this end, a structural similarity search of the four hit molecules in the docking set was carried out on the ‘clean’ and ‘in stock’ subset of the ZINC15 database, comprising ~6 million molecules. Any molecules showing a Tanimoto similarity value of >0.3 to any of the four structures of interest was included. This low threshold for Tanimoto similarity was intended to narrow the search space but without being overly restrictive of the available chemical landscape, yielding a dataset of ~9,000 compounds that composed the prospective ‘evaluation set’. The distribution of this evaluation set in terms of the predicting binding energies is shown in Supplementary Fig. 4a.

Different machine learning models were initially trialed against the docking scores calculated for the evaluation set as a test of the project feasibility, and these models were then tuned on the much smaller aggregation dataset. The best-performing setup, the RFR–GPR stacked model, was then trained on the whole aggregation dataset and used to predict the top set of molecules (see ‘Machine learning implementation’ section in Supplementary Information, and Supplementary Figs. 1, 5 and 6). For this work, the *t*_1/2_ for the light seeding assay was used as the metric of potency to be used in machine learning because of its robustness. For comparison, the amplification rate is more susceptible to small fluctuations in the slope of the aggregation fluorescence trace^23^ (Supplementary Fig. 7). Molecules that achieved a *t*_1/2_ twofold greater than that of the negative control under standard assay conditions (Methods) were classed as potent^45^. The algorithm was run repeatedly from different random starting states and those molecules that appeared in the top 100 ranked molecules more than 50% of the time (64 molecules) were chosen for purchase (first iteration). In this first iteration, there was an inherent bias toward the structure of molecule 69 in the dataset given the relative population sizes (Supplementary Fig. 2a), but with the caveat that many of these structures were only loosely related to the parent (Tanimoto similarity <0.4). Many of the potent molecules came from this group, suggesting chemical departures from the parent structure.

**Fig. 2:**
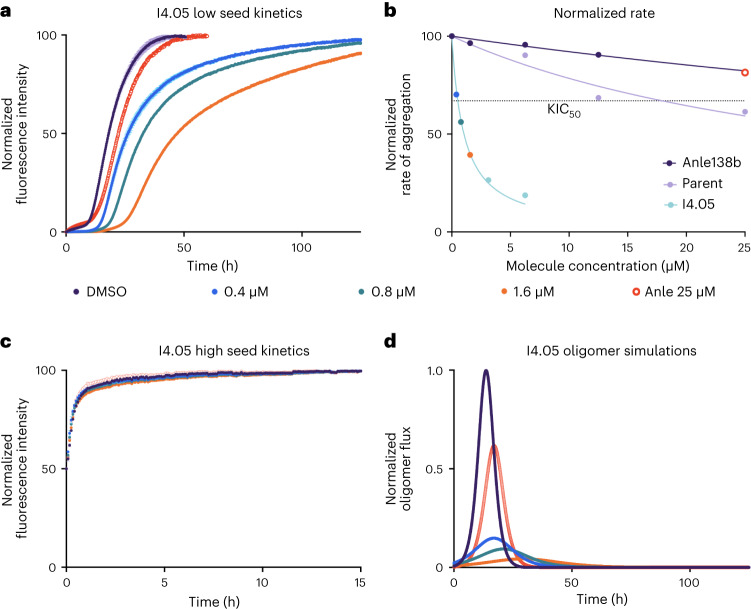
Performance comparison of a molecule from the iterative learning (I4.05) versus an αS aggregation inhibitor currently in clinical trials (Anle-138b). **a**, Kinetic traces of a 10 µM solution of αS with 25 nM seeds at pH 4.8, 37 °C in the presence of molecule or 1% DMSO (*n* = 3 replicates; central measure, mean; error, standard deviation (s.d.)). During the initial screening, except for iteration 4, all molecules were screened at 2.5 molar equivalents (25 µM), and potent molecules were then taken for further validation at lower concentrations: 0.4 µM (blue), 0.8 µM (teal), 1.6 µM (orange) with Anle-138b at 25 µM for comparison (red circles). The 1% DMSO negative control is shown in purple. Molecule I4.05 is shown as an example. The endpoints are normalized to the αS monomer concentration at the end of the experiment, which was detected via the Pierce BCA Protein Assay at *t* = 125 h. **b**, Approximate rate of reaction (taken as 1/*t*_1/2_, normalized between 0 and 100; central measure, mean) in the presence of three different molecules, Anle-138b (purple), parent structure 69 (lilac) and I4.05 (blue). The KIC_50_ of I4.05 is indicated by the intersection of the fit (blue) and the horizontal dotted line. **c**, High-seeded experiments (5 µM seeds, all other conditions match **a**, *n* = 3 replicates; central measure, mean; error, s.d.) were also carried out to observe any effects on the elongation rate and enable oligomer flux calculations in combination with the secondary nucleation rate derived from **a**. **d**, Oligomer flux calculations for I4.05 versus the clinical trial molecule Anle-138b using the rates derived from both **a** and **c**.

The dynamic range within the aggregation dataset in terms of potency was large, in that a majority of the molecules had no effect on aggregation, while initial docking hits exhibited relative *t*_1/2_ of up to four to five times that of the negative control (limited by the length of the experimental run) at 25 μM. Molecules then found via machine learning produced a relative *t*_1/2_ of ~4–5 at up to eightfold lower concentration (3.12 μM, 0.3:1 molecule:protein) than that carried out in the initial screening (25 μM, 2.5:1 molecule:protein). This compares favorably with previous molecular matter tested in a less aggressive seeded aggregation assay such as the flavone derivatives, apigenin, baicalein, scutellarein and morin, which achieved relative *t*_1/2_ of 1–2 at a stoichiometry of 0.5:1 molecule:protein^9^. Anle-138b^12^ is another example of a well-characterized small molecule inhibitor, which was also taken into clinical trials, whose relative *t*_1/2_ is 1.22 (Fig. 2) at a ratio of 2.5:1 molecule:protein in the assay used in this work, which is lower than any of the molecules discovered using the strategy employed here.

After the first iteration, the compound data were pooled together to extend the training set and a further two iterations were carried out with the updated model, adding the resultant data to the training set at each iteration. This was followed by a fourth and final iteration trained on low dose (3.12 μM) data of all the previously obtained molecules. Example kinetic traces for a molecule from the fourth iteration are shown in Fig. 2a. The molecules are labeled according to iteration number and lead identifier within that iteration. For example I4.05 is the fifth potent lead (05) within iteration 4 (I4). The dose-dependent potency in the aggregation assay was investigated (Fig. 2a and Supplementary Fig. 8) with all potent lead molecules exhibiting substoichiometric potency. For comparison, Anle-138b is also shown.

Figure 2b shows an approximate overall rate of aggregation at different concentrations of I4.05, Anle-138b and the parent molecule. This approximate rate was taken as 1/*t*_1/2_, and fitted to a Hill slope. A kinetic inhibitory constant (KIC_50_) was then derived. This is the concentration of molecule at which the *t*_1/2_ is increased by 50% with respect to the control, as defined previously^45^. The KIC_50_ values for the leads were in the range of 0.5–5 μM, which compare favorably with the parent of the lead molecules (molecule 69) and Anle-138b which have extrapolated KIC_50_ values of 18.2 μM and 36.4 μM, respectively. I4.05 had a KIC_50_ value of 0.52 µM with 95% confidence limits of 0.45 µM and 0.59 µM.

The elongation rate was largely unaffected in the presence of molecules at any concentration (Fig. 2c). This was expected given the designed mechanism of action of the small molecules. It was also reassuring, since compounds that inhibit elongation may increase the population of oligomers^45^, which are considered the most damaging of the aggregate species in vivo^7,8^. Then, using the amplification and elongation rates derived from Fig. 2a,c, the oligomer population over time was calculated^9^ (Methods). These calculations are shown in Fig. 2d for I4.05 and Supplementary Fig. 8 for the rest of the leads. All potent leads demonstrated a dose-dependent delay and reduction of the oligomer peak. Across all metrics, I4.05 performed better than Anle-138b and the parent molecule at substoichiometric ratios, as do all of the leads obtained in previous iterations (Supplementary Figs. 8 and 9).

**Fig. 3:**
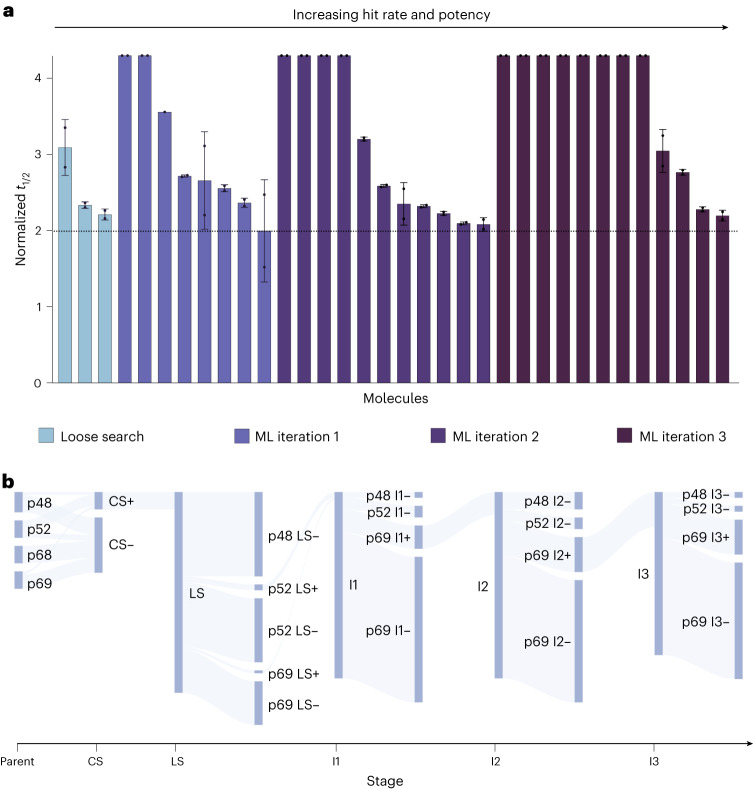
Results of the iterations of the machine learning drug discovery approach. **a**, Normalized *t*_1/2_ for the potent leads at 25 µM from the different stages: loose search, iteration 1, iteration 2 and iteration 3 (*n* = 2 replicates; central measure, mean; error, standard deviation). The horizontal dotted line indicates the boundary for potent lead classification, which was normalized *t*_1/2_ = 2. For the loose search, 69 molecules were tested, while for iterations 1, 2 and 3, the number of molecules tested was 64, 64 and 56, respectively. Note that the most potent molecules exhibited complete inhibition of aggregation over the timescale observed, so the normalized *t*_1/2_ is presented as the whole duration of the experiment. **b**, Flow of potent molecules (+) and negatives (−) in the project starting from the close search (CS), moving to the loose search (LS) and then iterations 1, 2, and 3 (I1, I2 and I3). Each branch is labeled with the molecule source (for example, p48). Attrition reached its highest point at the loose search before gradually improving with each subsequent iteration.

The aggregation data from the first three iterations are also shown in Fig. 3a. Of the 64 molecules from iteration 1, 8 were potent, representing an optimization rate of 12.5%, the second iteration showed a further increase, with 11 potent molecules, representing a 17.2% optimization rate, and the third iteration, with 12 potent molecules, had an optimization rate of 21.4%. These optimization rates represent an order of magnitude improvement over high-throughput screening hit rates (<1%)^46^ and, remarkably, an overall 40% improvement over the combined similarity search optimization rates, which removed the most likely lead candidates. The potency of the machine learning leads was also higher on average than those identified by the similarity searches (Supplementary Fig. 10a), without compromising the CNS-MPO scores (Supplementary Fig. 10b). The flow of molecules derived from each parent in terms of positives and negatives over the course of the project is illustrated in Fig. 3b. The accumulated training data from all stages of the project for all molecules in terms of half-time distribution is shown in Supplementary Fig. 4b,c.

Given that αS aggregation and toxicity has also been linked to membrane interactions^7,47^ a parallel investigation was carried out with a lipid-induced aggregation assay (Supplementary Fig. 11), which was used as a validation of the molecules rather than for machine learning optimization. The tested lead molecules also showed strong efficacy in this assay. A further test of these molecules in a spontaneous αS aggregation assay, without induction via pre-seeding or shaking, also exhibited strong potency^48^.

### Analysis of the chemical space explored by machine learning

The chemical space explored by the machine learning approach was inspected via dimensionality reduction techniques, including principal component analysis, *t*-distributed stochastic neighbor embedding^49^ and uniform manifold approximation and projection (UMAP)^50^ (Methods) to investigate how the model was prioritizing molecules (Supplementary Fig. 12). The relative positioning of the training points and the parents within the chemical space is shown in Supplementary Fig. 13a. The stacked RFR–GPR model assigned low uncertainty to areas of the chemical space proximal to the observed data, and the corresponding acquirement priority mirrored this when trained on the aggregation data (Supplementary Fig. 13b–d). Supplementary Fig. 13 also illustrates how the uncertainty weighting could be altered during the ranking, depending on how conservative a prediction was required. A drawback to a high uncertainty penalty was that the model remained in the chemical space it was confident in, while a lower uncertainty penalty ensured reasonable confidence of potent lead acquirement while still exploring the chemical space.

The changes in similarity of the potent leads to the parent structures are shown in Supplementary Fig. 14. The similarity of the molecules to their parent structure dropped for all structures at successive stages of the investigation, reaching its lowest point at the iterations of the machine learning approach. The more potent leads mostly retained the central ring and benzene substituent of molecule 69 albeit with the addition of polar groups to the benzene ring, but featured alterations to the rest of the scaffold. For example, from iteration 1, I1.01 replaced the fused ring substructure of molecule 69 with a single substituted benzene ring, while I1.02 replaced it with a substituted furan ring, and subsequent iterations saw more complexity introduced. These changes were reflected in the Tanimoto similarity values, which were at the lower end of what was permitted in the evaluation set, 0.3 being the cutoff. It was evident from this result that parts of the substructure were important to retain for potency, which the model did effectively while also identifying alterations in the rest of the scaffold that enhanced the potency considerably beyond that of the parent.

The observation that component 3, the quantitative structure activity relationship (QSAR) model, converges on the structures from two areas of the UMAP space related to structure 69 was encouraging. It suggested the model was learning useful information and not selecting at random. While we have not tested a random set of molecules due to prohibitive resource cost, we do note that, if a random selection of molecules were taken from the accumulated training data from all stages of the project, its optimization rate (11%) would be lower than that of iterations 1, 2 and 3 on average. Though performance improves with additional data, the QSAR performance in terms of *R*^2^ remains modest (Supplementary Fig. 1), but this is in part due to sparsity of training data. We would anticipate improvement if this approach could be implemented at medium scale with correspondingly more complex QSAR models, and we have an indication of this from trials of the this model set up against the docking scores of the evaluation set, where performance in terms of *R*^2^ score is threefold higher for a slightly larger dataset (Supplementary Fig. 6).

Next, an investigation was carried out to identify what structural information the latent vectors were encoding. Variational autoencoders are generally not built to ensure that their latent space dimensions are human interpretable, making this a challenge. The decoding of a variational autoencoder is also not deterministic, preventing facile analysis of the feature space based on single perturbation approaches of the input features and observing changes to decoded structures. Instead, hierarchical clustering was carried out on the latent vectors, followed by SHAP^51^ (Shapley additive explanations) clustering for comparison (Supplementary Fig. 15). While the former differentiated groups based on large changes in any dimension, clustering based on SHAP dimensions ensured that clusters were created only on the basis of features relevant to the prediction problem at hand. Latent space dimensions that have a large range of values had a large effect on the latent space clustering, regardless of whether these dimensions were important predictors of molecular potency. Using SHAP values, on the other hand, meant that latent space dimensions that had little effect on the model prediction were mapped to values close to zero, and therefore had a much smaller influence on the clustering. This resulted in clusters which were relevant to the prediction task. This strategy was suggested by the authors of SHAP and was recently used in the context of identifying subgroups of coronavirus disease 2019 symptoms^52^.

Supplementary Fig. 15 shows two-dimensional UMAP representations of the tested molecules, with the latent vector clustering indicated by color and the SHAP clustering indicated by shape. From the UMAP representation, we note that the SHAP clustering identified clusters more effectively than the hierarchical clustering. The SHAP values for each feature show the importance of that feature in the interpretation of potency, and this in turn could be used to identify which substructures within the molecules are relevant for potency by observing the structures that recurred in each cluster. For example, Supplementary Fig. 15 shows the top dimensions of each SHAP cluster, revealing that dimension 24 at least partly encoded for the key substructure 3,5-pyrazolidinedione, which was present in every molecule in cluster α and a proportion of cluster β. This confirmed the hypothesis previously put forward^30^ that, in a junction tree variational autoencoder, the latent space encoding preserved the key features of each molecule. Molecules that were clustered together shared many molecular substructures in common.

### Measurement of binding affinity

**Fig. 4:**
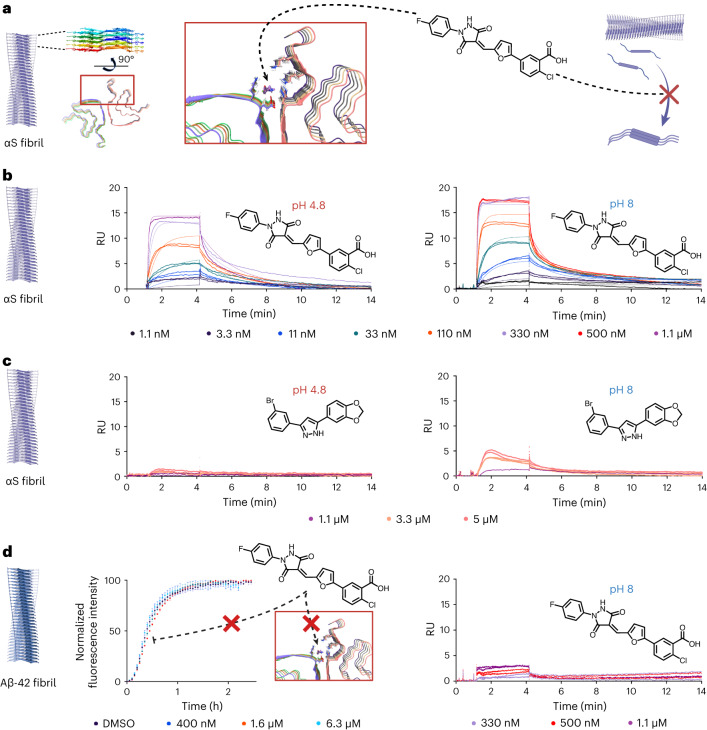
Molecule binding to αS fibrils. **a**, A schematic representation of small molecule binding to the target binding pocket on the αS fibril. **b**, SPR response curves for different concentrations of I4.05, at pH 4.8 and pH 8, binding to αS fibrils generated by a seeded assay, with the corresponding molecular structure. Raw data (points) and the corresponding fits (solid lines) for each molecule concentration are shown (*n* = 2 replicates). Response units (RU) are shown on the *y*-axes. The αS fibrils were immobilized at a concentration of 2,000 pg mm^−^^2^ on a CM5 Cytivia chip. The fits correspond to a 1:1 kinetic binding model, which yielded a *K*_D_ of 68 nM (*k*_a_ = 1.936 ± 0.007 × 10^5^ M^−1^ s^−1^, *k*_d_ = 1.315 ± 0.003 × 10^−2^ s^−1^) at pH 4.8 and 13 nM at pH 8 (*k*_a_ = 5.879 ± 0.024 × 10^5^ M^−1^ s^−1^, *k*_d_ = 0.781 ± 0.002 × 10^−2^ s^−1^). Error: standard error of the mean (s.e.m.). **c**, SPR response curves for different concentrations of Anle-138b. Raw data (points) for each molecule concentration are shown (*n* = 2 replicates). Accurate fits at pH 4.8 could not be obtained. At pH 8 a 1:1 kinetic binding model yielded an approximate *K*_D_ of 8.1 μM (*k*_a_ = 0.0359 ± 0.0005 × 10^5^ M^−1^ s^−1^, *k*_d_ = 2.90 ± 0.02 × 10^−2^ s^−1^). Error: s.e.m. **d**, Seeded kinetics (40 nM seed, *n* = 2 replicates; central measure, mean; error, standard deviation) and SPR response curves (*n* = 2 replicates) for 2 μM Aβ42 in the presence of 1% DMSO or different concentrations of I4.05. I4.05 is unable to effectively inhibit Aβ42 secondary nucleation or bind to Aβ42 fibrils. The Aβ42 fibrils were immobilized at a concentration of 2,000 pg mm^−^^2^ on a CM5 Cytivia chip.

A series of validation experiments were carried out on the most potent leads from the machine learning iterations. We first tested the binding to fibrils using surface plasmon resonance (SPR; Methods) under different buffer conditions. The results for molecule I4.05 versus Anle-138b are shown in Fig. 4. The proposed mechanism of action is the binding of molecules to the fibrils thereby blocking nucleation sites for further aggregation. Support for this mechanism of action comes from the observations that the molecules function at substoichiometric ratios, discounting monomer interactions, and also show negligible effect on elongation. Covalent interactions can also be discounted, as no mass change is observed of the αS monomer by mass spectrometry. The large effect observed in an assay that isolates secondary nucleation as the dominant mechanism implies that the molecules are specifically affecting this step, and the substoichiometry implies that the molecules must be interacting with the fibrils that are present in nanomolar monomer equivalents at the start of the aggregation.

Proof of binding and evidence for this potential mechanism are shown by SPR in Fig. 4. Figure 4a shows a schematic representation of molecule binding to the binding pocket targeted during the initial docking simulation. Figure 4b shows SPR response curves for a concentration range between 0.3 nM and 1.1 μM of I4.05 binding to immobilized αS fibrils, while Fig. 4c shows the same experiment utilizing Anle-138b from 1.1 μM to 5 μM. The binding was tested under the conditions of the αS secondary nucleation assay (pH 4.8), and also at pH 8, allowing direct comparison to the secondary nucleation conditions of Aβ42, which were tested as a negative control in Fig. 4d. αS is highly charged at neutral pH and has an isoelectric point (pI) of 4.7 (ref. ^53^). It therefore requires a pH in this region to render the protein uncharged in order to aggregate on an experimentally accessible timescale under quiescent conditions, whereas Aβ42 is highly aggregation prone and requires higher pH to prevent it aggregating too rapidly^45^. At both pH values, I4.05 exhibited binding to αS fibrils, with kinetic fits giving *K*_D_ values of 68 nM at the lower pH and 13 nM at the higher pH. The data for Anle-138b showed no response for pH 4.8, and so no *K*_D_ could be obtained, while at pH 8 an approximate *K*_D_ of 8.1 µM was obtained. It was evident that the two orders of magnitude improvement in KIC_50_ of I4.05 compared to Anle-138b was matched by a similar degree of improvement in terms of binding efficacy. Figure 4d shows that I4.05 has no effect on the seeded aggregation of Aβ42, nor does it bind effectively to Aβ42 fibrils, which suggests that this molecule is not a promiscuous aggregation inhibitor between different amyloidogenic proteins.

### Inhibition of aggregation using brain-derived seeds

**Fig. 5:**
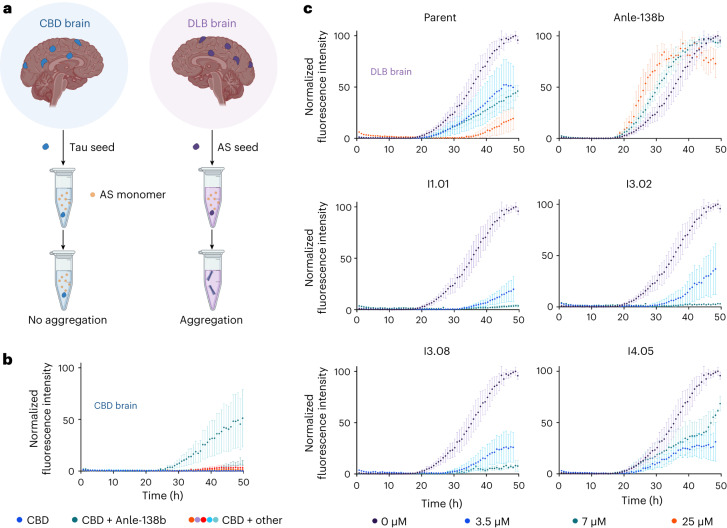
RT-QuIC brain seeding assay. **a**, Schematic representation of the RT-QuIC assay. Aggregates derived from the brain tissue of patients suffering with DLB were used to induce αS aggregation. Samples from brains of patients with CBD were used as a negative control. **b**, Kinetic traces of a 7 µM solution of αS in the presence of CBD seeds (pH 8, 42 °C, shaking at 400 rpm with 1 min intervals, *n* = 4 replicates; central measure, mean; error, standard deviation (s.d.)). CBD samples were 1% DMSO (blue), 7 µM Anle-138b (teal), parent (orange), I1.01 (purple), I3.02 (red), I3.08 (turquoise) and I4.05 (light blue). Anle-138b, in teal, induces aggregation under this condition. **c**, Kinetic traces of a 7 µM solution of αS in the presence of DLB seeds (*n* = 4 replicates; error, s.d.; all other conditions match **b**). The DLB samples were 1% DMSO (purple), 3.5 µM molecule (blue), 7 µM molecule (teal) and 25 µM molecule (orange). Anle-138b again appears to accelerate rather than inhibit aggregation.

While this result was encouraging, with the recent determination of the pathological αS fibril structure^54^, it became clear that the recombinant in vitro fibril structure we had employed for computational and experimental work was different to that found in the brains of patients with PD. To test whether these molecules might work against patient-derived fibrils, these molecules were tested in a real-time quaking-induced conversion (RT-QuIC) seed amplification assay (Fig. 5) that employs brain samples from patients suffering with dementia with Lewy bodies (DLB). The dominant fibril structure identified in DLB was found to match the dominant structure observed in PD^54^.

The RT-QuIC assay was initially introduced as a diagnostic assay^55,56^, showing distinct aggregation curves in the presence of brain material derived from different pathologies^57^. In this case, we use it to test the ability for these molecules to slow the aggregation of αS induced by DLB brain material. As a negative control, samples from patients with a tauopathy (corticobasal degeneration, CBD) were also used, as these did not induce αS aggregation as no αS seeds were present (Fig. 5a,b). No aggregation was observed in the CBD samples over the timescale observed except for Anle-138b, which accelerated aggregation under this condition. This unusual behavior may be due to Anle-138b’s reportedly low solubility^12^. The conditions are different to those initially screened, as this assay was carried out at pH 8 and utilized shaking to accelerate seeded aggregation. This is a more challenging paradigm for the molecules to function in as multiple aggregation processes occur in tandem^41^. In addition to secondary nucleation from the fibril surfaces, fragmentation of the fibrils induced via shaking results in more fibril ends for elongation, which in turn provides more fibril surface for secondary nucleation.

Despite these challenges, and the different fibril structure present, the lead molecules still function well in inhibiting aggregation, and still at substoichiometric ratios (Fig. 5c). There was a clear improvement for the leads over Anle-138b, which again appeared to accelerate aggregation, and the parent molecule, although the ranking of the leads in terms of efficacy is altered compared to the screening assay. To understand these results we note that there is a similarity in the binding pockets in the structures 6CU7 (recombinant) and 8A9L (brain derived) (Supplementary Fig. 16). We currently do not know whether this similarity is serendipitous, but binding pockets with similar features can also be observed via cryogenice electron microscopy in the multiple system atrophy (MSA) type I and MSA type II fibril folds as well as the Lewy fold, with an unresolved species bound within the pocket^54^.

To account for differences in brain samples and also investigate potential efficacy against MSA-derived brain material, we tested a single concentration of the same selection of molecules against three neuropathologically confirmed MSA brain samples (Supplementary Fig. 17a,c) and two further DLB brain samples (Supplementary Fig. 17a,d). As a further negative control, a sample with no seed or brain material was tested, to determine the degree of spontaneous nucleation in the absence of an inducer (Supplementary Fig. 17b). Aggregation in this negative control was effectively inhibited by all the potent ML molecules, given that αS was likely to assume the 6CU7 polymorph in this condition, and not by Anle-138b, which accelerated aggregation. It should be noted that the CBD samples are the better negative control for RT-QuIC, as all brain samples contain tissue matrix components that may sequester αS and reduce its aggregation. The unseeded sample began aggregation at ~40–50 h, whereas CBD samples did not exhibit aggregation over a span of 80 h (Supplementary Fig. 17e). Fibrils present in DLB and MSA samples were able to counteract this effect. For the other DLB and MSA samples, broadly similar trends were observed to those shown in Fig. 5. The ML molecules did appear more efficacious against MSA samples (Supplementary Fig. 17c), perhaps because the MSA pocket more closely matches that of the targeted 6CU7 polymorph (four flanking lysines around a histidine residue) compared to the 8A9L polymorph found in PD and DLB (four flanking lysines around a tyrosine residue) as shown in Supplementary Fig. 16. The behavior of Anle-138b was variable as, where the ML-derived molecules inhibited aggregation to some extent across all examples, Anle-138b either had no effect (unseeded and MSA samples 1 and 2) or induced (CBD sample, MSA sample 3 and DLB sample 1) or mildly inhibited aggregation (DLB samples 2 and 3).

### Oligomer quantification by microfluidic free-flow electrophoresis

**Fig. 6:**
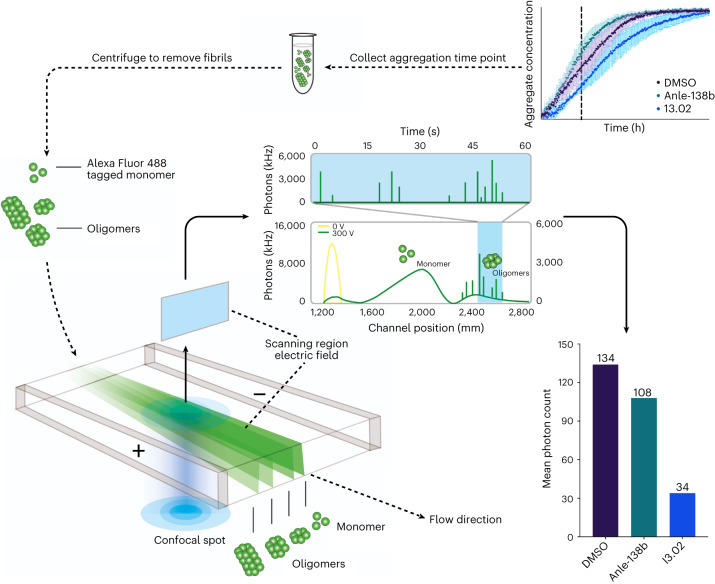
Quantification of αS oligomers using μFFE. Top right: αS labeled with AlexaFluor 488 (100 µM, pH 7.4, 37 °C, cycles of 5 min shaking at 200 rpm and 1 min rest, *n* = 4 replicates; error, standard deviation) was supplemented with 0.5 µM seed and 1% DMSO (purple) or 50 µM Anle-138b (teal) or I3.02 (blue) in 1% DMSO. Anle-138b slightly accelerates aggregation under these conditions, where fragmentation mechanisms may again play a role due to shaking, while I3.02 slows it down. Samples were extracted at 9 h from the time course of aggregation and centrifuged to remove fibrils from the mixture, leaving only αS monomers and soluble oligomeric species for analysis via μFFE. Bottom left: schematic representation of the μFFE approach, showing the AlexaFluor 488-labeled αS oligomeric mixture undergoing μFFE. The direction of fluid flow is shown by arrows. The differential deflection of the electric field allows the monomer population to be separated from the oligomer population during analysis. Middle and bottom right: analysis of the aggregate populations detected in each sample. The mean number of photons emitted, proportional to particle number and size, is plotted on the *y* axis of the bar plot for each sample. The average number of photons emitted per particle is indicated in the inset.

Having observed that molecule I3.02 was the most broadly effective in the RT-QuIC assay, an investigation was carried out to directly measure the oligomeric species formed during the reaction. This was achieved using microfluidic free-flow electrophoresis (µFFE)^58^, a technique optimized using similar conditions to that used in the RT-QuIC assay, albeit at higher αS concentration (100 µM). The results of this are shown in Fig. 6. Aggregation time courses were tracked using AlexaFluor 488 labeled N122C αS rather than ThT. Figure 6 shows a schematic of the approach, where samples were extracted from an aggregation time course, centrifuged to remove insoluble aggregates, and finally submitted to µFFE. The degree of deflection and the photon count of each particle are proportional to the size and charge of the biomolecule. The former allows the separation of monomers from oligomers and the latter gives a measure of the number and size of the oligomers at a particular time point in the presence of different inhibitors. Oligomer electrophoretic mobility (*μ*_o_) for an oligomer composed of *n*_m_ monomer units is proportional to oligomer charge (*q*_o_) and inversely proportional to oligomer hydrodynamic radius (*r*_o_) and so can be described by^58^

1 $${\mu }_{{\mathrm{o}}}\propto \frac{{q}_{{\mathrm{o}}}}{{r}_{{\mathrm{o}}}}\propto \frac{{{n}_{{\mathrm{m}}}}^{v}}{{r}_{{\mathrm{o}}}}$$

where *v* is a scaling exponent linking *q*_o_ with *n*_m_. Approximating the oligomers as spherical species yields^58^

2 $${\mu }_{o}\propto \frac{{{n}_{{\mathrm{m}}}}^{v}}{{r}_{{\mathrm{m}}}{{n}_{{\mathrm{m}}}}^{\frac{1}{3}}}=\frac{{{n}_{{\mathrm{m}}}}^{{v}^{* }}}{{r}_{{\mathrm{m}}}}$$

where the oligomer electrophoretic mobility is defined only in terms of the monomer number (*n*_m_) and hydrodynamic radius (*r*_m_), and the scaling exponent v* = *v* − 1/3. Samples were extracted at the *t*_1/2_ of the negative control (1% dimethyl sulfoxide (DMSO)) and the results are shown in Fig. 6. Anle-138b dosing resulted in a smaller population of large aggregates, as may be expected from the slight acceleration in the aggregation observed in the fluorescence values, while I3.02 reduced both the size and the number of oligomers present in comparison to the DMSO control. The ranking of these inhibitors was further validated in a subsequent study of oligomer levels using solid state nanopores combined with DNA nanostructure tagging^59^.

## Discussion

The identification of inhibitors of αS aggregation based on chemical kinetics approaches has advanced to the point that specific steps in the aggregation process, including primary nucleation and secondary nucleation, can be targeted in a reproducible way^9,28,29^. The mechanism targeted in this work is the surface-catalyzed secondary nucleation step, which is responsible for the autocatalytic proliferation of αS fibrils. In a recent initial report, initial hit molecules identified via docking simulations were shown to bind competitively with αS monomers along specific sites on the surface of αS fibrils^23,24,60^. Specific rate measures and other aggregation metrics were derived from these experiments allowing quantitative and reliable comparisons between molecules in terms of structure–activity relationship and offering metrics to optimize structures of interest^9,45^. This has been augmented with tests against diseased brain material and detailed, experimental fibril binding and oligomer flux analyses.

The aim of this work was to develop a machine learning approach to drug discovery for protein aggregation diseases that could improve both the optimization rate of the in vitro assays employed and provide novel chemical matter more efficiently than conventional approaches. The optimization rate of the approach was an over 20-fold improvement over typical high-throughput screening hit rates (~0–1%)^61^. These structures also represent discoveries that could not have been obtained by staying close in chemical space to the parent structure, as would have been dictated by similarity search approaches. There were ~4,000 molecules in the evaluation set that had Tanimoto similarity values in the same range as these leads, and all of these would potentially have had to be screened to locate these molecules using similarity searches alone. This was demonstrated by the looser similarity search approach which exhibited a comparatively poor optimization rate (4%) despite more conservative structural alterations to the parent hits than were observed in the ML predicted molecules. The machine learning method was therefore able to supply a degree of novelty as well as an improved optimization rate.

A limitation of this approach is the requirement to select molecules from a pre-existing library. To resolve this limitation generative modelling combined with reinforcement learning has been applied in a parallel project to remove the need for a library to screen from^44,62^. A second limitation is the focus on one assay metric of interest as a learning parameter. Addressing this limitation will involve future work on multiparameter optimization, which is a challenging area in rapid development^63–65,66^. Another topic of great interest in drug discovery approaches based on machine learning besides potency prediction is the prediction of pharmacokinetics and toxicity^67,68^. It could be possible to achieve this multiparameter optimization utilizing multiple models in parallel and then employing a joint ranking metric, or architectures that screen for individual metrics in series. This has been previously demonstrated but primarily with chemical properties such as clogP and QED rather than experimental results^63–65^. The molecules in this work were derived from a set that passed CNS MPO criteria in the initial docking simulation, and so the CNS MPO metrics of the whole aggregation inhibitor set are relatively favorable with most hit molecules exceeding the common cutoff value of 4 (ref. ^39^) (Supplementary Fig. 10b).

It would have been preferable to begin this approach using seeds derived from relevant pathological brain material, but this was not possible, as neither structures nor samples for these were available at the start of this study. Nonetheless, we have demonstrated that these molecules still function against disease-relevant inducers, probably because of the degree of commonality between the binding sites of the fibril polymorphs. The complete loss of function against another aggregation prone protein, Aβ42, does however suggest specific functionality against αS.

## Conclusions

The results that we have presented illustrate a drug discovery approach that involves an iterative structure-based machine learning strategy to generate potent protein aggregation inhibitors. The resulting molecules offer a large improvement in potency over the parent molecule and clinical trial molecules and represent a major structural departure from them. We anticipate that using machine learning approaches of the type described here could be of considerable benefit to researchers working in the field of protein misfolding diseases, and indeed early-stage drug discovery research in general.

## Methods

### Compounds and chemicals

Compounds were purchased from MolPort or Mcule and prepared in DMSO to a stock of 5 mM. All chemicals used were purchased at the highest purity available.

### Recombinant αS expression

Recombinant αS was purified on the basis of previously described methods^25,41,69^. The plasmid pT7-7 encoding human αS was transformed into BL21 (DE3) competent cells. Following transformation, the competent cells were grown in 6L 2xYT medium in the presence of ampicillin (100 μg ml^−1^). Cells were induced with isopropyl β-d-1-thiogalactopyranoside, grown overnight at 28 °C and then collected by centrifugation in a Beckman Avanti JXN-26 centrifuge with a JLA-8.1000 rotor at 6,240 rcf (Beckman Coulter). The cell pellet was resuspended in 10 mM Tris, pH 8.0, 1 mM ethylenediaminetetraacetic acid (EDTA), 1 mM phenylmethylsulfonyl fluoride and lysed by sonication. The cell suspension was boiled for 20 min at 85 °C and centrifuged at 39,000 rcf with a JA-25.5 rotor (Beckman Coulter). Streptomycin sulfate was added to the supernatant to a final concentration of 10 mg ml^−1^ and the mixture was stirred for 15 min at 4 °C. After centrifugation at 39,000 rcf, the supernatant was taken with an addition of 0.36 g ml^−1^ ammonium sulfate. The solution was stirred for 30 min at 4 °C and centrifuged again at 39,000 rcf. The pellet was resuspended in 25 mM Tris, pH 7.7, and the suspension was dialyzed overnight in the same buffer. Ion-exchange chromatography was then performed using a Q Sepharose HP column of buffer A (25 mM Tris, pH 7.7) and buffer B (25 mM Tris, pH 7.7, 1.5 M NaCl). The fractions containing αS were loaded onto a HiLoad 26/600 Superdex 75 pg Size Exclusion Chromatography column, and the protein (~60 ml @ 200 µM) was eluted into the required buffer. The protein concentration was determined spectrophotometrically using *ε*_280_ = 5,600 M^−1^ cm^−1^. The cysteine-containing variant (N122C) of αS was purified by the same protocol, with the addition of 3 mM dithiothreitol to all buffers.

### Labeling of αS

αS protein was fluorophore-labeled to enable visualization by fluorescence microscopy. To remove dithiothreitol, cysteine variants of αS were buffer exchanged into phosphate-buffered saline (PBS) or sodium phosphate buffer by use of P10 desalting columns packed with Sephadex G25 matrix (GE Healthcare). The protein was then incubated with an excess of AlexaFluor 488 dye with maleimide moieties (Thermo Fisher Scientific) (overnight, 4 °C on a rolling system) at a molar ratio of 1:1.5 (protein to dye). The labeling mixture was loaded onto a Superdex 200 16/600 (GE Healthcare) and eluted in PBS buffer at 20 °C, to separate the labeled protein from free dye. The concentration of the labeled protein was estimated by the absorbance of the fluorophores, assuming a 1:1 labeling stoichiometry (AlexaFluor 488: 72,000 M^−1^ cm^−1^ at 495 nm).

### αS seed fibril preparation

αS fibril seeds were produced as described previously^25,41^. Samples of αS (700 µM) were incubated in 20 mM phosphate buffer (pH 6.5) for 72 h at 40 °C and stirred at 1,500 rpm with a Teflon bar on an RCT Basic Heat Plate (IKA). Fibrils were then diluted to 200 µM, aliquoted and flash frozen in liquid N_2_, and finally stored at −80 °C. For the use of kinetic experiments, the 200 µM fibril stock was thawed, and sonicated for 15 s using a tip sonicator (Bandelin, Sonopuls HD 2070), using 10% maximum power and a 50% cycle.

### Measurement of αS aggregation kinetics

αS was injected into a Superdex 75 10/300 GL column (GE Healthcare) at a flow rate of 0.5 ml min^−1^ and eluted in 20 mM sodium phosphate buffer (pH 4.8) supplemented with 1 mM EDTA. The obtained monomer was diluted in buffer to a desired concentration and supplemented with 50 µM ThT and preformed αS fibril seeds. The molecules (or DMSO alone) were then added at the desired concentration to a final DMSO concentration of 1% (v/v). Samples were prepared in low-binding Eppendorf tubes, and then pipetted into a 96-well half-area, black/clear flat-bottom polystyrene non binding surface microplate (Corning 3881), 150 µl per well. The assay was then initiated by placing the microplate at 37 °C under quiescent conditions in a plate reader (FLUOstar Omega, BMG Labtech). The ThT fluorescence was measured through the bottom of the plate with a 440 nm excitation filter and a 480 nm emission filter. After centrifugation at 2,350 rcf to remove aggregates the monomer concentration was measured via the Pierce BCA Protein Assay Kit according to the manufacturer’s protocol.

For the lipid induced assay, small unilamellar vesicles containing 1,2-dimyristoyl-*sn*-glycero-3-phospho-l-serine (Avanti Polar Lipids), were prepared from chloroform solutions of the lipids as described previously^69^. Briefly, the lipid mixture was evaporated under a stream of nitrogen gas and then dried thoroughly under vacuum to yield a thin lipid film. The dried thin film was re-hydrated by adding aqueous buffer (20 mM sodium phosphate, pH 6.5, and 1 mM EDTA) at a concentration of 1 mM and heating to 40 °C for 2 h while stirring at 1,500 rpm with a Teflon bar on an RCT Basic Heat Plate (IKA). Small unilamellar vesicles were obtained using several cycles of freeze–thawing followed by extrusion through membranes with 200 nm diameter pores (Avanti Polar Lipids). αS was prepared as above. Kinetic conditions were 20 µM αS, 100 µM 1,2-dimyristoyl-*sn*-glycero-3-phospho-l-serine, 50 µM ThT, 30 °C; all other conditions remained the same as above.

Transmission electron microscopy (TEM) imaging of the fibrils produced at the end of the light seeded aggregation reaction (Supplementary Fig. 18) was used to verify fibrils were produced

### Determination of the αS elongation rate constant

In the presence of high concentrations of seeds (approximately micromolar), the aggregation of αS is dominated by the elongation of the added seeds^25,41^. Under these conditions where other microscopic processes are negligible, the aggregation kinetics for αS can be described by^9,23,25^

$${\left.\frac{{{\mathrm{d}}M}(t)}{{{\mathrm{d}}t}}\right|}_{t=0}=2{k}_{+}P\left(0\right)m(0)$$

where *M*(*t*) is the fibril mass concentration at time *t*, *P*(0) is the initial number of fibrils, *m*(0) is the initial monomer concentration, and *k*_*+*_ is the rate of fibril elongation. In this case, by fitting a line to the early time points of the aggregation reaction as observed by ThT kinetics, 2*k*_*+*_*P*(*0*)*m*(*0*) can be calculated for αS in the absence and presence of the compounds. Subsequently, the elongation rate in the presence of compounds is expressed as a normalized reduction as compared to the elongation rate in the absence of compounds (1% DMSO).

### Determination of the αS amplification rate constant

In the presence of low concentrations of seeds (approximately nanomolar), the fibril mass fraction, *M*(*t*), over time was described using a generalized logistic function to the normalized aggregation data^9,70^

$$\frac{M(t)}{{m}_{{{\mathrm{tot}}}}}=1-\frac{1}{{\left[1+\frac{a}{c}{e}^{\,\kappa t}\right]}^{c}}$$

where *m*_tot_ denotes the total concentration of αS monomers. The parameters *a* and *c* are defined as

$$a=\frac{{\lambda }^{2}}{2{\kappa }^{2}}$$

$$c=\sqrt{\frac{2}{{n}_{2}({n}_{2}+1)}}.$$

The parameters $\lambda$ and $\kappa$ represent combinations for the effective rate constants for primary and secondary nucleation, respectively, and are defined as^70^

$$\lambda =\sqrt{2{k}_{+}{k}_{{\mathrm{n}}}{m}_{{{\mathrm{tot}}}}^{{n}_{{\mathrm{c}}}}}$$

and

$$\kappa =\sqrt{2{k}_{+}{k}_{2}{m}_{{{\mathrm{tot}}}}^{{n}_{2}+1}},$$

where *k*_n_ and *k*_2_ denote the rate constants for primary and secondary nucleation, respectively, and *n*_c_ and *n*_2_ denote the reaction orders of primary and secondary nucleation, respectively. In this case, *n*_c_ was fixed at 0.3 for the fitting of all data (corresponding to a reaction order of *n*_2_ = 4), and *k*_2_, the amplification rate, is expressed as a normalized reduction for αS in the presence of the compounds as compared to in its absence (1% DMSO).

### Determination of the αS oligomer flux over time

The theoretical prediction of the reactive flux toward oligomers over time was calculated as^9,70^

$$\phi \left(t\right)=\frac{1}{{r}_{+}}{\rm{\cdot }}\left[\frac{m(0)}{m(t)}{\rm{\cdot }}\frac{{{\mathrm{d}}}^{2}M}{{\mathrm{d}}{t}^{2}}+\frac{1}{m(0)}{\left(\frac{m(0)}{m(t)}{\rm{\cdot }}\frac{{{\mathrm{d}}M}(t)}{{{\mathrm{d}}t}}\right)}^{2}\right]$$

where *r*_*+*_ *=* *2k*_*+*_*m*(*0*) is the apparent elongation rate constant extracted as described earlier, and *m*(*0*) refers to the total concentration of monomers at the start of the reaction.

### Recombinant Aβ42 expression

The recombinant Aβ42 peptide (MDAEFRHDSGY EVHHQKLVFF AEDVGSNKGA IIGLMVGGVV IA), here called Aβ42, was expressed in the *Escherichia coli* BL21 Gold (DE3) strain (Stratagene) and purified as described previously. Briefly, the purification procedure involved sonication of *E. coli* cells, dissolution of inclusion bodies in 8 M urea, and ion exchange in batch mode on diethylaminoethyl cellulose resin followed by lyophylization. The lyophilized fractions were further purified using Superdex 75 HR 26/60 column (GE Healthcare) and eluates were analyzed using sodium dodecyl sulfate polyacrylamide gel electrophoresis for the presence of the desired peptide product. The fractions containing the recombinant peptide were combined, frozen using liquid nitrogen, and lyophilized again.

### Aβ42 aggregation kinetics and fibril preparation

Solutions of monomeric Aβ42 were prepared by dissolving the lyophilized Aβ42 peptide in 6 M guanidinium hydrocholoride (GuHCl). Monomeric forms were purified from potential oligomeric species and salt using a Superdex 75 10/300 GL column (GE Healthcare) at a flow rate of 0.5 ml min^−1^, and were eluted in 20 mM sodium phosphate buffer, pH 8 supplemented with 200 µM EDTA and 0.02% NaN_3_. The center of the peak was collected and the peptide concentration was determined from the absorbance of the integrated peak area using *ε*_280_ = 1,490 l mol^−1^ cm^−1^. The obtained monomer was diluted with buffer to the desired concentration and supplemented with 20 μM ThT from a 2 mM stock. Each sample was then pipetted into multiple wells of a 96-well half-area, low-binding, clear-bottom and polyethylene glycol-coated plate (Corning 3881), 80 µl per well, in the absence and the presence of different molar-equivalents of small molecules (1% DMSO). Assays were initiated by placing the 96-well plate at 37 °C under quiescent conditions in a plate reader (Fluostar Omega, Fluostar Optima or Fluostar Galaxy, BMGLabtech). The ThT fluorescence was measured through the bottom of the plate using a 440 nm excitation filter and a 480 nm emission filter. Fibrils were extracted directly from wells and used on the day for SPR experiments.

### Machine learning

#### Junction tree neural network variational autoencoder

The autoencoder^30^ was pretrained on a library of 250,000 compounds^31^, and was implemented using a pip installable version^71^ in addition to torch (1.10.0), RDKit (2020.09.1), MolVS (0.1.1) and scipy (1.5.2). Any molecules that contained substructures the autoencoder could not represent (that is, that fell outside the substructure vocabulary of the pretrained model) were excluded.

#### Prediction module

All coding was carried out in Python 3. Scikit-learn (0.24.1)^72^ implementations of the GPR, RFR, LR and MLP methods were tested in various combinations, and the results are shown in Supplementary Information. For data handling, calculations and graph visualization the following software and packages were used: pandas (1.2.4)^73^, seaborn (0.11.1)^74^, matplotlib (3.3.4)^75^, numpy (1.20.1)^76^, scipy (1.6.2)^77^, fbpca (1.0), umap-learn (0.3.10)^50^, Multicore-TSNE (0.1)^49^ and GraphPad Prism (9.1.2). Cross validation and benchmarking were also carried out for each model using scikit-learn built in functions and is described in Results.

#### SHAP and latent space clustering

To compute the SHAP values, we used the SHAP python library^51^. The pretrained random-forest model was loaded, and a SHAP explainer object was created and provided with the latent representation for the top 100 highest predicted molecules. This allowed for the identification of dimensions important to the prediction of high potency molecules. The full testing set derived from the ZINC dataset was also used to differentiate between dimensions important to distinguish high-potency molecules from low-potency molecules versus dimensions important to distinguish high-potency molecules between themselves. This resulted in a global interpretation of the model, encompassing all data points passed to the explainer object. The resultant plots were generated using SHAP built-in plot functions. The sklearn library hierarchical clustering method was used to cluster latent vectors for comparison, with initial cluster number set to 7 (ref. ^78^).

### SPR

All work was carried out using Biacore T200 at 25 °C. CM5 chips were activated by flowing 0.01 M N-hydroxysuccinimide, 0.4 M 1-Ethyl-3-diaminopropyl carbodiimide at a flow rate of 10 µl min^−1^ for 7 min over two lanes. Preformed αS or Aβ42 fibrils (derived from the endpoints of low seeded aggregation reactions) at a concentration of 1 µM in sodium acetate (10 mM, pH 4.0) were injected onto a single lane in 60 s bursts at 5 µl min^−1^ until a response of 2,000 units was reached. Both lanes were then deactivated using a 7-min injection of ethanolamine (1 M, pH 8.5) at 10 µl min^−1^, and the reference lane signal was subtracted from the active lane. Different small molecule concentrations were then flowed over both lanes in a pyramidal arrangement in duplicate with blank subtraction (association time 3 min, dissociation time 10 min). The running buffer was sodium phosphate (20 mM, 1 mM EDTA, variable pH) with 1% DMSO. Fitting was carried out on Biacore T200 Evaluation Software, version 3.2, using a 1:1 binding model with the refractive index set to a constant value of 0 response units.

### Brain tissue samples and compliance with ethical standards

Deidentified post-mortem brain samples were obtained from sources indicated in Supplementary Table 2. As samples were obtained from deceased, deidentified, consenting individuals, no further ethical approval was required.

### Preparation of human brain tissue homogenates

Deidentified post-mortem human brain specimens used in the RT-QuIC assay are referenced in Supplementary Table 2. These specimens were obtained from the NIH Brain & Tissue repository-California, Human Brain & Spinal Fluid Resource Centre, VA West Los Angeles Medical Center, Los Angeles, California, which is supported in part by National Institutes of Health and the US Department of Veterans Affairs. Assay samples were prepared as 10% (wt/vol) brain homogenates in ice-cold PBS (pH 7.0) using 1 mm zirconia beads (BioSpec, cat no. 11079110z) in a Bead Mill 24 (Fisher Scientific). Subsequent dilutions of each brain homogenate (10^−1^ to 10^−5^) for testing in the RT-QuIC assay were prepared in 1× PBS (pH 7.0).

### αSyn RT-QuIC protocol

RT-QuIC assay for DLB samples were performed using the recombinant αSyn K23Q substrate purified using a two-step chromatography protocol described previously (PMID: 29422107). For testing MSA samples, wild-type αSyn recombinant substrate was purified using anion-exchange and size exclusion chromatography as described in PMID: 15939304 with minor modifications. The wild-type protein expressing pET21a-αS plasmid was a gift from Michael J Fox Foundation MJFF (Addgene plasmid no. 51486; http://n2t.net/addgene:51486; RRID: Addgene_51486). RT-QuIC assay was performed using black, clear-bottom 96-well plates (Nalgene Nunc International) preloaded with six silica beads (1 mm diameter, OPS Diagnostics). Seeding was induced by addition of 2 μl of 10^−4^ (with respect to solid brain tissue) dilutions of DLB, MSA or CBD (control) brain homogenates in quadruplicate wells containing 98 μl of the reaction buffer (40 mM phosphate buffer; pH 8.0 and 170 mM NaCl) supplemented with 6 μM (0.1 mg ml^−1^) αSyn K23Q substrate (prefiltered through 100 kDa molecular weight cutoff filter, Pall Corporation, cat. no. OD100C34) and 10 μM ThT. After seeding, reaction plates were covered with a sealer film (Nalgene Nunc International) and incubated at 42 °C in a fluorescence plate reader (BMG FLUOstar Omega) with 1 min shake–rest cycles (400 rpm double orbital) for 50–90 h as indicated in the figures. ThT fluorescence (*λ*_excitation_ = 450 ± 10 nm and *λ*_emission_ = 480 ± 10 nm) was measured at 45 min intervals).

### µFFE

#### Microfluidic device fabrication

Devices were designed using AutoCAD (24.3) software (Autodesk) and photolithographic masks printed on acetate transparencies (Micro Lithography Services). Polydimethylsiloxane devices were produced on SU-8 molds fabricated via photolithographic processes as described elsewhere^79,80^ with ultraviolet exposure performed with custom-built light-emitting diode-based apparatus^81^. Following development of the molds, feature heights were verified by profilometer (Dektak, Bruker) and polydimethylsiloxane (Dow Corning, primer and base mixed in 1:10 ratio) applied and degased before baking at 65 °C for 1.5 h. Devices were cut from the molds and holes for tubing connection (0.75 mm) and electrode insertion (1.5 mm) were created with biopsy punches, the devices were cleaned by application of Scotch tape and sonication in isopropanol (5 min). After oven drying, devices were bonded to glass slides using an oxygen plasma. Before use, devices were rendered hydrophilic via prolonged exposure to oxygen plasma^82^.

#### μFFE device operation

Liquid-electrode microchip free-flow electrophoresis (μFFE) devices were used^83^. Briefly, fluids were introduced to the device by PTFE tubing, 0.012″ inner diameter × 0.030″ outer diameter (Cole-Parmer) from glass syringes (Gas Tight, Hamilton) driven by syringe pumps (Cetoni neMESYS). μFFE experiments were conducted with auxiliary buffer, electrolyte, monomer reference and sample flow rates of 1,000, 200, 140 and 10 μl h^−1^, respectively, for 15× reduction in buffer salt concentration for samples in PBS buffer.

Potentials were applied by a programmable benchtop power supply (Elektro-Automatik EA-PS 9500-06) via bent syringe tips inserted into the electrolyte outlets. Experiments were performed on a custom-built single-molecule confocal fluorescence spectroscopy setup equipped with a 488 nm wavelength laser beam (Cobolt 06-MLD 488 nm 200 mW diode laser, Cobolt). Photons were detected using a time-correlated single photon counting module (TimeHarp 260 PICO, PicoQuant) with a time resolution of 25 ps.

#### Aggregation kinetics and sample extraction

AlexaFluor 488-labeled αS (100 μM) was supplemented with seed (0.5 μM) under shaking (200 rpm) at 37 °C, PBS pH 7.4 and either 1% DMSO or 50 μM molecule in 1% DMSO. Samples were extracted at the *t*_1/2_ of the DMSO sample (9 h). Fibrils were removed by centrifugation (21,130 rcf, 10 min, 25 °C) and the supernatant was then subjected to μFFE. For AlexaFluor 488-labeled oligomeric mixtures, auxiliary buffer composed of 15× diluted PBS buffer, supplemented with 0.05% v/v Tween-20. Using a custom-written script, single-molecule events were recorded as discrete events using a Lee filter of 4 from the acquired photon stream as fluorescence bursts with 0.05 μs of the maximum inter-photon time and containing 30 photons minimum. Using these parameters, the single-molecule bursts and their intensities were reported as a function of device position, which could be later converted to an apparent electrophoretic mobility. Oligomer bursts were distinctly characterized by a higher photon intensity detected per molecule and a higher electrophoretic mobility than monomeric protein.

### Mass spectrometry

Ten micromolar of preformed αS was incubated with 25 µM of molecule in 20 mM sodium phosphate buffer (pH 4.8) supplemented with 1 mM EDTA overnight under quiescent conditions at room temperature. The supernatant was removed for analysis using a Waters Xevo G2-S QTOF spectrometer (Waters Corporation).

### TEM

Ten micromolar αS samples were prepared and aggregated as described in the kinetic assay, without the addition of ThT. Samples were collected from the microplate at the end of the reaction (150 h) into low-binding Eppendorf tubes. They were then prepared on 300-mesh copper grid containing a continuous carbon support film (EM Resolutions) and stained with 2% uranyl acetate (wt/vol) for 40 s. The samples were imaged at 200 kV on a Thermo Scientific (FEI) Talos F200X G2 S/TEM (Yusuf Hamied Department of Chemistry Electron Microscopy Facility). TEM images were acquired using a Ceta 16M CMOS camera.

### Reporting summary

Further information on research design is available in the Nature Portfolio Reporting Summary linked to this article.

## Online content

Any methods, additional references, Nature Portfolio reporting summaries, source data, extended data, supplementary information, acknowledgements, peer review information; details of author contributions and competing interests; and statements of data and code availability are available at 10.1038/s41589-024-01580-x.

### Supplementary information

Supplementary Information Supplementary methods, Tables 1 and 2 and Figs. 1–18.

Reporting Summary

## Data Availability

The data that support the findings of this study are available within the main text and its Supplementary Information. Additional datasets can be found on the GitHub repository at https://github.com/rohorne07/Iterate.

## Code availability

Full code can be found on the GitHub repository at https://github.com/rohorne07/Iterate.
