# Supplementary material for: Discovery of potent inhibitors of α-synuclein aggregation using structure-based iterative learning
Source: Nat Chem Biol. 2024 Apr 17;20(5):634–45. doi: 10.1038/s41589-024-01580-x (PMC11062903; doi:10.1038/s41589-024-01580-x)
Supplement: Supplementary file 1 — Supplementary methods, Tables 1 and 2 and Figs. 1–18. [file 41589_2024_1580_MOESM1_ESM.pdf]

nature chemical biology

Article

https://doi.org/10.1038/s41589-024-01580-x

Discovery of potent inhibitors of α-synuclein aggregation using structure-based iterative learning

In the format provided by the authors and unedited

Table of Contents

Supplementary Methods 2
Table S1. 7
Table S2. 8
Figure S1. 12
Figure S2. 14
Figure S3. 16
Figure S4. 17
Figure S5. 18
Figure S6. 19
Figure S7. 20
Figure S8. 23
Figure S9. 25
Figure S10. 26
Figure S11. 27
Figure S12. 28
Figure S13. 29
Figure S14. 30
Figure S15. 31
Figure S16. 32
Figure S17. 33
Figure S18. 35
Supplementary References 36

Supplementary Methods

Docking and machine learning implementation

A full description of the initial docking approaches can be found in the previous work[1], using AutoDock Vina[2] and FRED[3] docking software, but is also explained in overview here. As described in the main text, the binding site encompassing residues His50−Lys58 and Thr72−Val77 on PDB 6CU7[4] was selected due to its propensity to form a pocket according to Fpocket[5] software and its simultaneous mid to low solubility according to CamSol[6] (**Figure S3**). Additionally, a key histidine residue in this site was predicted to protonate below the pH value where αS more readily aggregates (pH 5.8). A binding box was selected that had size 12 Å by 12 Å by 9 Å centred at 10.00 Å, 9.89 Å, 11.52 Å on the 6CU7 PDB, encompassing the site of interest. The target protein was left rigid, while the ligand was flexible, able to translate and rotate (including rotation of internal bonds). We prepared the target protein (added hydrogens) using Autodock tools. To increase the accuracy of the docking energy estimate, the exhaustiveness was increased compared to the default value of 8, to 20. 5 poses were output, and the best pose binding energy was selected as the binding energy label for that ligand. The choice of rigid target was made in order to decrease the computational cost of the high throughput screen of the 2 million compounds in phase 1.

Inspired by the increasing usage of consensus scoring, i.e combining multiple docking energy estimates by different docking programs, we performed docking of the 100,000 best binding molecules from AutoDock Vina, using FRED in phase 2. For each of the top 100,000 best AutoDock Vina ligands, we combine the ligand with the target into a single .pdb file, and from that supply the information of the ligand to Openeye's Spruce module to prepare an .oedu file that contains the grid position of the binding site. Then, the compound is bound to the target site and a single best pose and binding energy is output, that constitutes the FRED binding energy label for this compound. The top 10,000 are then clustered to obtain representative centroids for testing. The pipeline is modular, and it is possible to incorporate any type of docking software the user might choose. In this study we have used AutoDock Vina, which is a publicly available software that is efficient at scale, and FRED. AutoDock Vina is relatively computationally efficient at scale, and we chose to use FRED since the top scoring

pose prediction of FRED has been shown[7] to be able predict within 2Å of the native pose in 70% of examples tested. However, alternative open-source or free for academic use docking software such as rDock, LeDock and others can be used instead of FRED, with relatively little difference in performance as shown previously[8]. The performance of AutoDock Vina is comparable with other open-source software.

The code for testing the ML models on aggregation or docking data are available at https://github.com/rohorne07/Iterate. We initially tested the machine learning strategy on docking data (best R^2 ~0.6-0.7) before moving to experimental aggregation data (best R^2 ~0.2-0.3) to get an impression of the feasibility of the project, given the larger datasets available for the docking scores (**Figure S5** and **Figure S6**). The docking scores were calculated for the 'evaluation set', the in silico library that was used for iterative experimental screening in the main text. Both AutoDock Vina and FRED simulations were carried out on the evaluation set, giving binding scores for each molecule against the αS 6CU7 fibril structure pocket. The compound encoder was implemented as in Hie et al.[9] to obtain representations of all the molecules. The next sections briefly summarise the functioning and output of the prediction module.

Prediction module. The prediction module consisted of a shallow model designed to be appropriate for small datasets and easily applicable on standard hardware available for most laboratory workers over a short timescale. As a first line test Gaussian process regression (GPR) was employed alone, following Hie et al.[9] with training and testing carried out with cross validation on 4000 molecules from within the evaluation set. The metric used to evaluate performance in this case was the R^2 score, or coefficient of determination. This score measures the goodness of fit between a set of predictions and the ground truth values. This score ranges from 1, in a perfect fit, to arbitrarily negative values as a fit becomes worse, and is 0 when the predictions are equivalent to the expectation of the ground truth values of the training set[10]. This was compared with a naïve Bayes, which failed to score above 0 for any training set size on both docking and aggregation data.

The GPR kernel was initially the same as that utilised by Hie et al.[9] , i.e. a combination of a constant kernel and a radial basis function (RBF). Using these initial settings, R^2 scores of ~0.2 were obtained for the docking data. Hyperparameter optimisation

yielded only marginal improvements in this performance. A selection of other kernels was tested, and all models were optimised via hyperparameter tuning before implementation, but most did not offer an improvement in performance. The Matérn kernel, a generalisation of the RBF with an extra parameter controlling the smoothness of the function, did however show a marginal improvement. These flexible functions are the most likely to be able to fit shallow energy minima problems such as those encountered here. The R^2 scores were still low, especially for smaller training sets as would be available from experiment, but represented a viable starting point.

At this point a 2-layer model was applied. This reflected the strategy used by Hie et al. [9] in fitting a Gaussian process regressor (GPR) to the residuals of another model, in that case a multi-layer perceptron (MLP). An MLP did not show a dramatic improvement over the GPR alone both in that work or when tested with the docking scores here, however a random forest regressor (RFR) with stacked GPR did show a further improvement both in terms of the R^2 (~0.6-0.7) and the quality of the molecule sets predicted during the simulation, as can be seen in **Figure S6**.

This set up gave improved results in both R^2 and hit rate, while retaining an easy to implement and efficient model. The average Pearson's coefficient of correlation ranged between 0.25 and 0.3 for both the coupled (GPR+RFR) and uncoupled models (RFR alone), which while modest matched the values obtained by Hie et al. during their testing. RFR was more demanding computationally, but given the small size of the experimental training sets in this scenario this was not a hindrance.

A simulation was created to mimic how the experimental cycle of testing might work using the docking scores as a surrogate for aggregation data. In the simulation, a random subset of 100 molecules was selected and the model trained on these molecules and their binding scores. The resultant model was then used to predict binding scores for the remaining molecules and rank them using a combination of the predicted value and the associated uncertainty value. The top 100 were then selected and their binding scores added to the training set as would occur in the experimental scenario, and this process was repeated 10 times. The ideal scenario would be that molecule sets with improved mean binding energy relative to the mean of the evaluation set would be selected, and that selections would improve as the training

set expanded, and this is what is shown in **Figure S5** (though improvement is not drastic as further data is added, possibly due to the relative ease with which strong dockers are selected).

Different uncertainty penalties were tested during this process. We found that a low uncertainty penalty produced better results by removing the most overconfident predictions without placing too many limitations on the model. At the early stages most predictions with low uncertainty were those with predicted binding scores close to the mean of the training set. An excessive uncertainty penalty during these stages would cause the model to only predict molecules that it was confident in, which were also likely to be mild.

The same process was utilised using different parts of the molecular feature set (the latent vector consists of a tree vector representation of clusters within a molecule, plus a graph representation of the molecule), and it was found that GPR performance metrics were better when using the molecular graph alone compared with using the entire representation. In general, it is to be expected that fitting fewer features to a predicted value is easier for a regressor to achieve and so higher scores are obtained. However, a better average R^2 score across the data set does not necessarily lead to a better result in terms of the actual molecules picked, and we found using the full representation led to more hits being identified (**Figure S5**).

A snapshot of the results of this testing is shown in **Figures S5** and **S6**. **Figure S5** demonstrates 2 points: the performance was slightly improved using the Matérn kernel in place of the RBF kernel both in terms of overall hit selection and performance improvement with increasing training set size, and the full-length molecular representation gave a significant boost in terms of number of hits selected vs the truncated representation, despite lower R^2 scores. These results also provided some evidence that Gaussian process learning might work reasonably effectively even in this data sparse scenario albeit at a modest level. It was expected that fitting experimental data would prove more challenging, however, and so a boost in performance was sought for that would not compromise the simplicity of the model, through use of the coupled RFR-GPR model. Correlation values of 0.6-0.7 were obtained using this set up on docking energies and a large portion of the dataset (4000

molecules), and this fell to between 0.2 and 0.3 for the aggregation data (**Figure S1**), which while low was encouraging given the much smaller dataset and noisier data.

A

Model	Parameter	
	Parameter label	*Parameter value*
LR	fit_intercept	True
LR	copy_X	True
LR	n_jobs	1
LR	positive	False
MLP	hidden_layer_sizes	100
MLP	activation	relu
MLP	solver	adam
MLP	alpha	0.0001
MLP	batch_size	auto
MLP	learning_rate	constant
MLP	learning_rate_init	0.001
MLP	max_iter	200
MLP	tol	0.0001
MLP	warm_start	False
MLP	early_stopping	False
MLP	beta_1	0.9
MLP	beta_2	0.999
MLP	epsilon	1×10^{-8}
MLP	n_iter_no_change	10

LR = linear regressor, MLP = multi layer perceptron

B

Model	Parameters		
	Kernel (if applicable)	*Parameter label*	*Parameter value*
GP	Constant	length_scale	1.0
GP	Constant	length_scale_bounds	fixed
GP	RBF	length_scale	1.0
GP	RBF	length_scale_bounds	fixed
GP	Matern	length_scale	1.0
GP	Matern	length_scale_bounds	fixed
GP	Matern	nu	1.5
RF	n/a	n_estimators	950
RF	n/a	criterion	squared_error
RF	n/a	max_depth	50
RF	n/a	min_samples_split	2
RF	n/a	min_samples_leaf	2
RF	n/a	min_weight_fraction_leaf	0.0
RF	n/a	max_features	log2
RF	n/a	max_leaf_nodes	None
RF	n/a	min_impurity_decrease	0.0
RF	n/a	bootstrap	False
RF	n/a	oob_score	False
RF	n/a	warm_start	False
RF	n/a	max_samples	None

GP = Gaussian Process, RF = random forest, RBF = Radial Basis Function

Table S1. **Parameters used in QSAR model optimisation.** (**A**) Models such as LR and MLP were trialled with their default parameters either alone or in conjunction with a GP, but showed poor performance so were not further investigated. (**B**) GP and RF models were the best performing and so were subjected to hyperparameter optimisation via grid search cross validation using the R^2 score as the optimisation metric. The best performing parameters are shown. The performance of these models is shown in **Figure S1**.

Brain Sample	Source	Gender (M/F)	Age at Death (years)	Disease duration (years)	Postmortem interval (h)	Primary diagnosis	Additional diagnosis
DLB1	Ghetti	M	81	N.A.	20	Diffuse Lewy body disease	Senile changes, Cerebrovascular disease
DLB2	BSFRC	M	70	6	N.A.	Lewy body Dementia	Senile changes, Cerebrovascular disease
DLB3	BSFRC	M	75	4	N.A.	Lewy body Dementia	Senile changes, Cerebrovascular disease
MSA1	BSFRC	F	62	N.A.	N.A.	Multiple system atrophy	N.A.
MSA2	Ghetti	F	71	N.A.	N.A.	Multiple system atrophy	Senile changes, Cerebrovascular disease
MSA3	Ghetti	M	52	N.A.	3	Multiple system atrophy	Senile changes, Cerebrovascular disease
CBD	Ghetti	F	51	10	9.6	Corticobasal Degeneration	N.A.

Table S2. Clinical and neuropathological characteristics of synucleinopathy and non-synucleinopathy brain tissue samples used in the study.

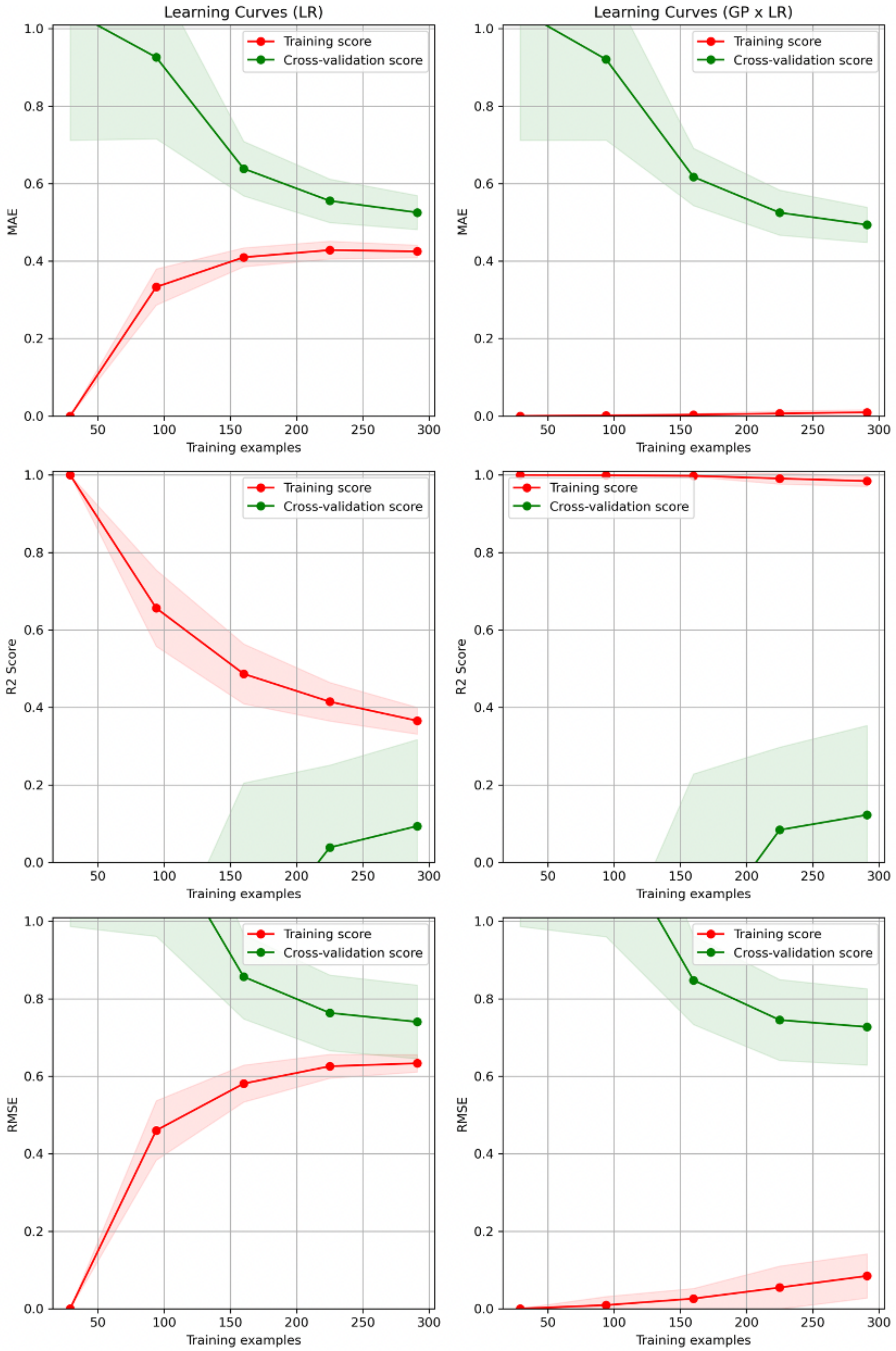
Learning Curves (LR)
Learning Curves (GP x LR)
Training score
Cross-validation score
MAE
R2 Score
RMSE
Training examples
0.0
0.2
0.4
0.6
0.8
1.0
50
100
150
200
250
300

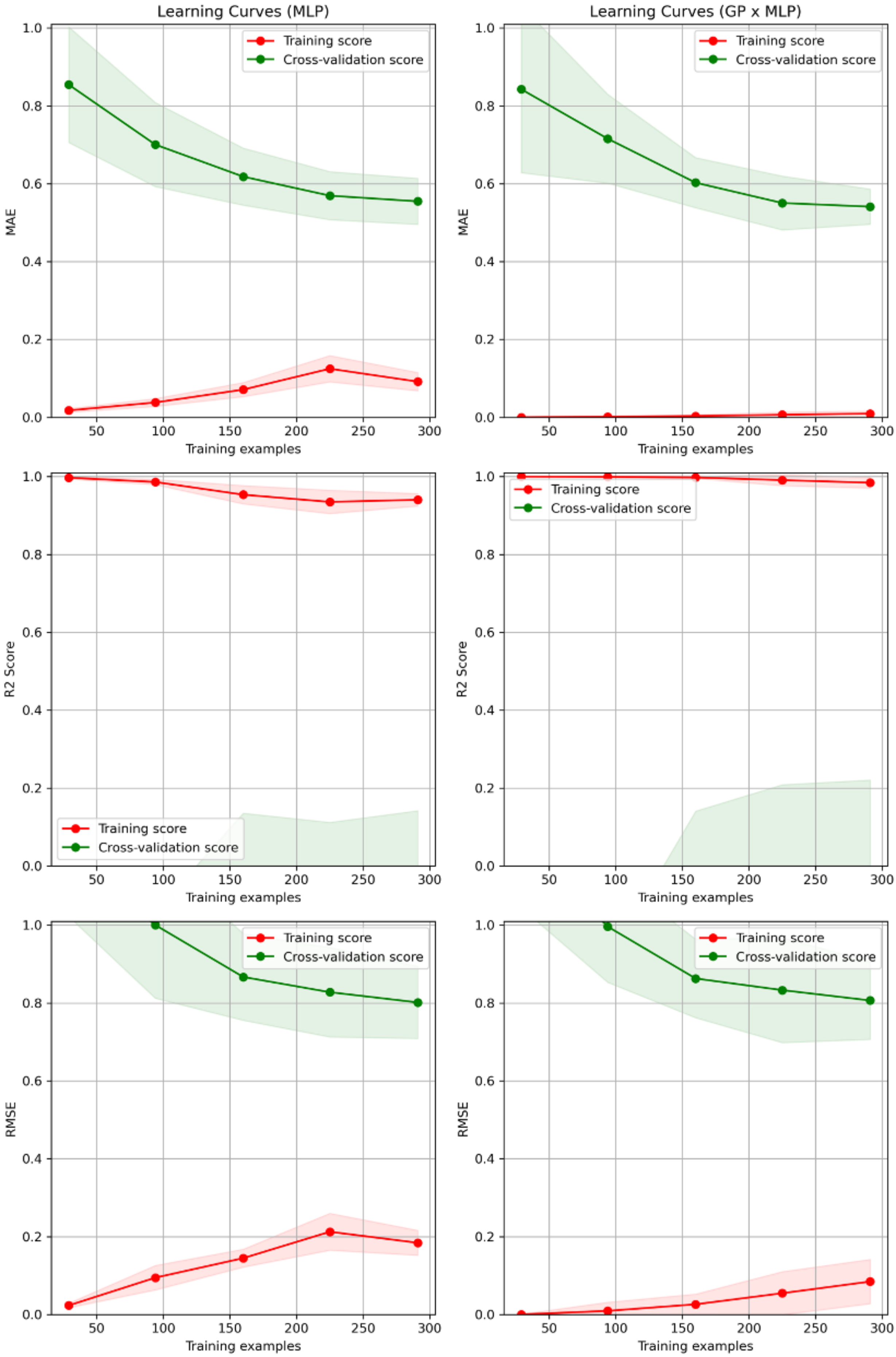
Learning Curves (MLP)
Learning Curves (GP x MLP)
Training score
Cross-validation score
MAE
R2 Score
RMSE
Training examples
0.0
0.2
0.4
0.6
0.8
1.0
50
100
150
200
250
300

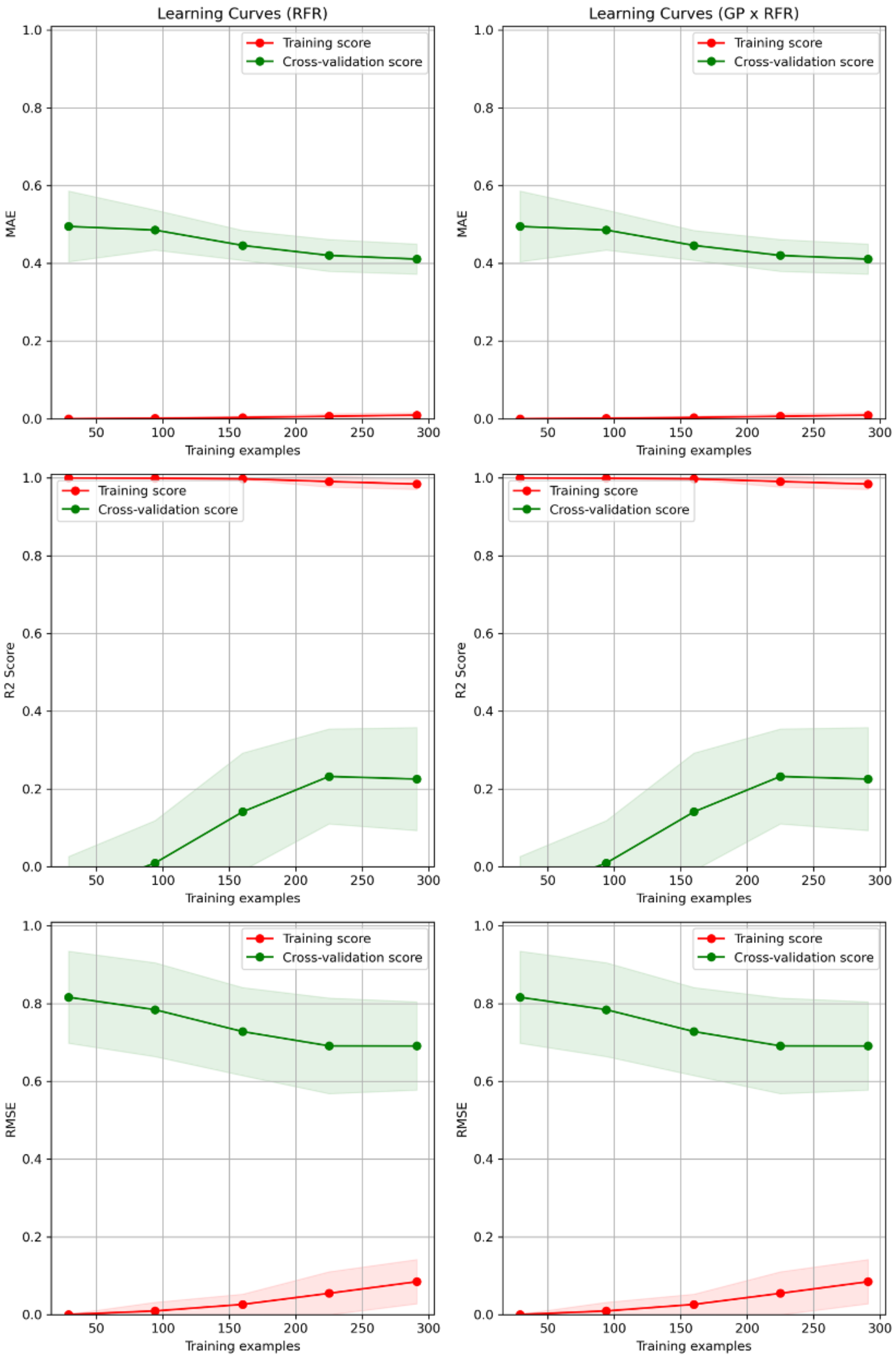
Learning Curves (RFR)
Learning Curves (GP x RFR)
Training score
Cross-validation score
MAE
R2 Score
RMSE
Training examples
0.0
0.2
0.4
0.6
0.8
1.0
50
100
150
200
250
300

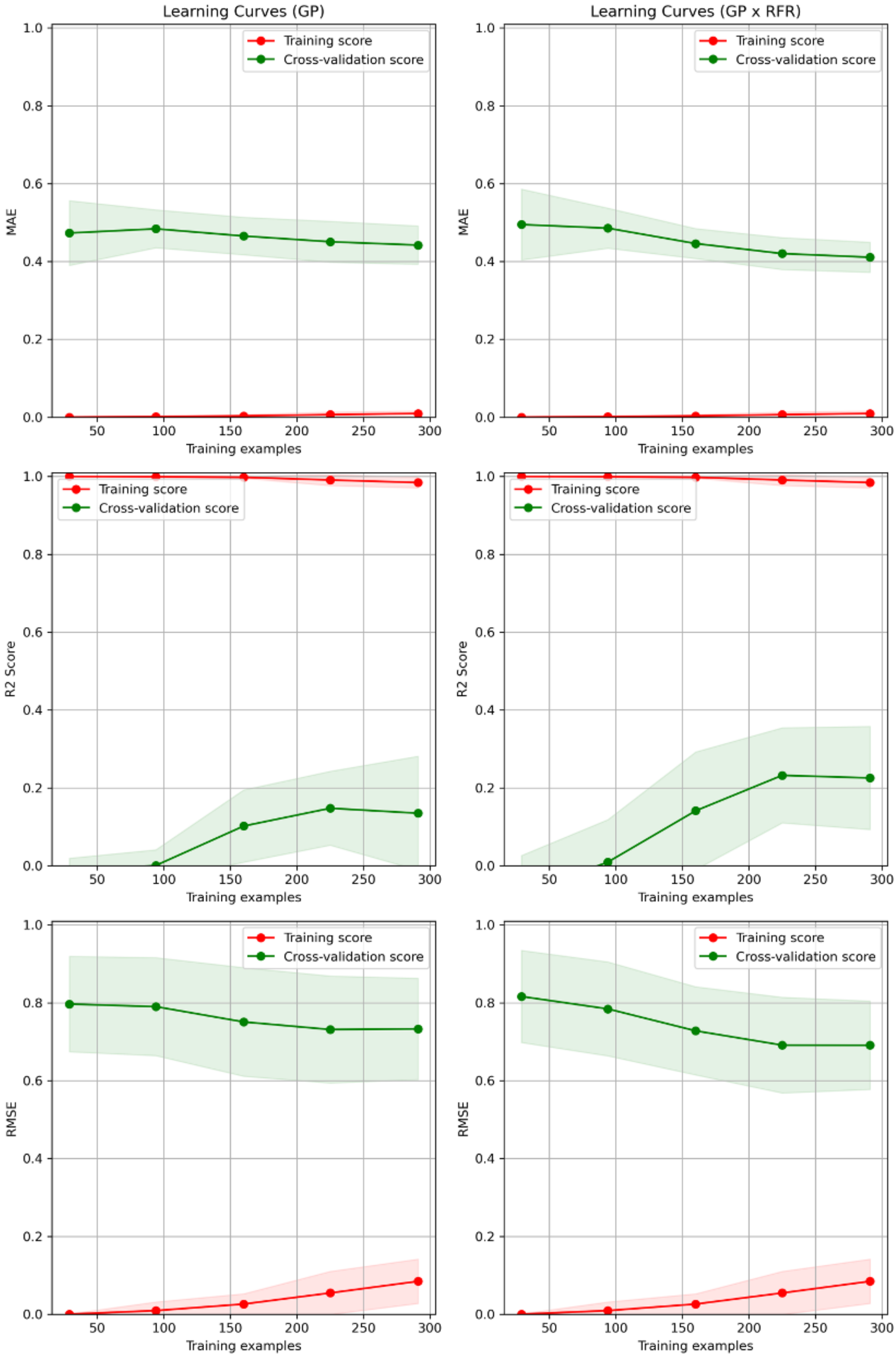

Figure S1. MAE, RMSE and R^2 for different models trained on the latent features

of the variational autoencoder and the aggregation data. The y-axis reports the respective scoring metric, and the x-axis the number of molecules included in the training set, of a total sample of 360 molecules (central measure=mean, error=SD). In each case, the performance of the model in isolation is shown in the left column, while the performance of the model when used in tandem with the GPR fitted to the residuals of the first model is shown in the right column. The labels are as follows: LR = linear regressor, GP = Gaussian process, MLP = multilayer perceptron, RFR = random forest regressor. Model parameters were chosen using a grid search of possible parameters while cross validating on 5 stratified K folds of the aggregation data, and selecting the parameters that gave the best performance in terms of R^2 score. The parameters for the models shown here are displayed in **Table S1**.

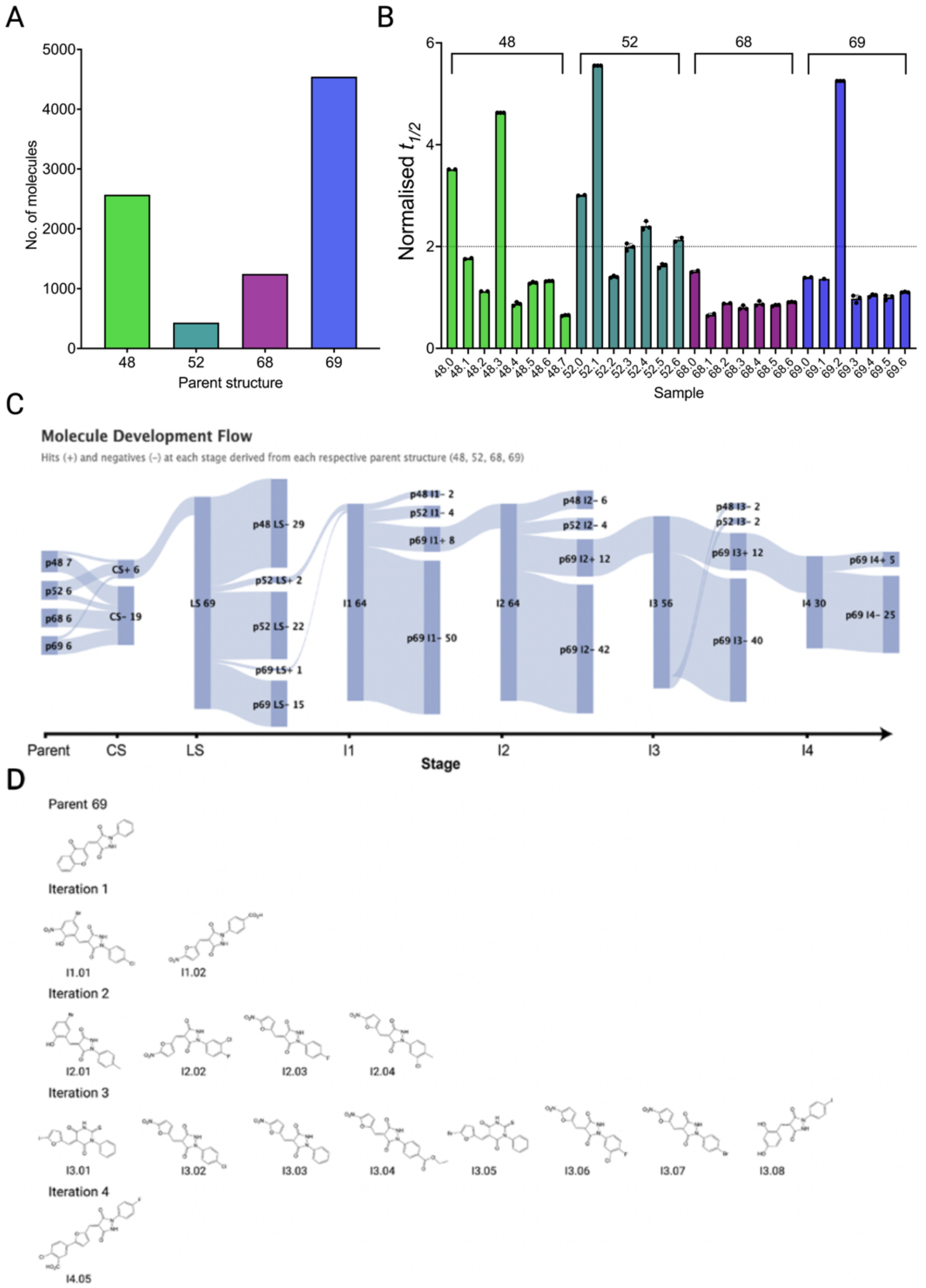

Figure S2. Summary of the molecules described in this work. (A) Number of molecules derived from 1 of the 4 docking hits (48, 52, 68, 69) within the evaluation set (see **Figure 1**). There were more structures derived from molecules 69 and 48 compared with molecules 68 and 52. **(B)** Normalized half time of aggregation ($t_{1/2}$) for the 25 molecules in the close similarity docking set (25 µM, n=2 replicates, central measure=mean, error=SD), i.e. those closely related (Tanimoto similarity > 0.5) to the 4 molecules in the docking set (labelled as 48.0, 52.0, 68.0 and 69.0 on the x-axis). Leads were defined as molecules that more than double $t_{1/2}$, as indicated by the horizontal line that marks 2 times the half time in the absence of the molecules. Some derivatives of molecules 48, 52 and 69 showed good potency, in particular 48.3, 52.1

and 69.2, but these effects were outstripped by future leads such as I4.05 which yielded the same effects at 10-fold lower concentration. **(C)** Flow chart of molecule leads (+) and negatives (-) in the project starting from the close search (CS), moving to the loose search (LS) then iterations 1, 2, 3 and 4 (I1, I2, I3, I4). Each branch is labelled with the molecule source (e.g. parent 48 = p48) whether it was a lead or a negative, and the number of molecules in the branch. Attrition reached its highest point at the loose search before gradually improving with each subsequent iteration. Iteration 4 is included but not directly comparable as a model was trained on the lower dose inhibitory data for this step. **(D)** Structures of the most potent hits/leads at each stage, which flatlined aggregation at 25 μM, all of which were derived from p69. The structures gradually converged as the core pyrazolidine-3,5-dione structure and RHS aromatic ring were largely retained (with some exceptions for ring expanded derivatives in iteration 3) with addition of electron withdrawing groups to the benzene ring. The LHS was altered more significantly, replacing the parent bicyclic system with substituted furans, which were further elaborated in iteration 4 with an additional benzoic acid group.

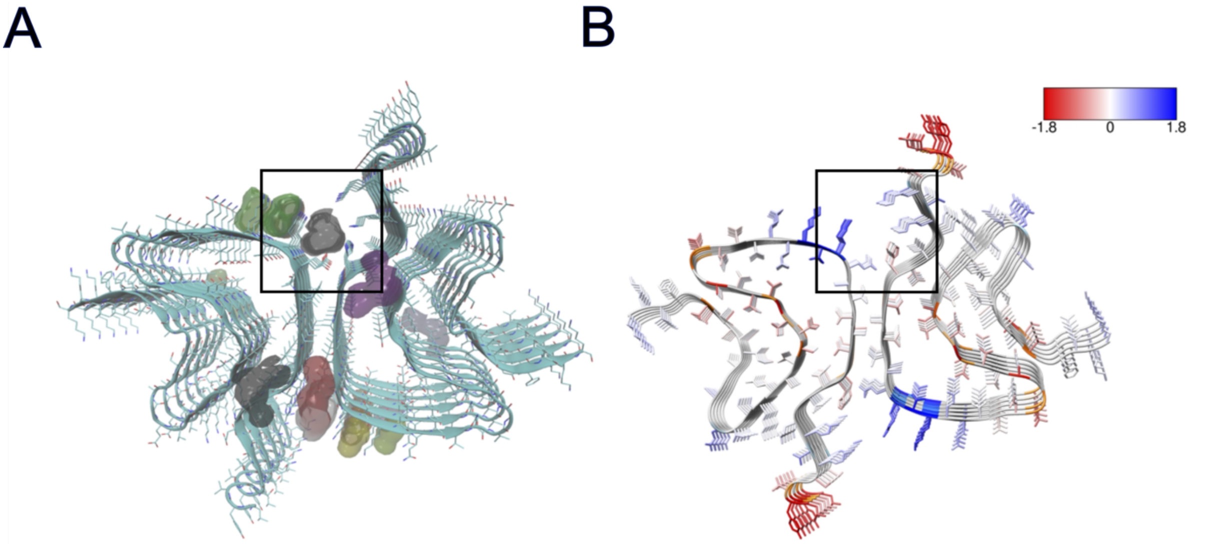

Figure S3. Volume and solubility based binding site prediction on polymorph 6CU7[4]. **(A)** Cavity based binding site prediction using Fpocket[5]. **(B)** Solubility based binding site prediction using CamSol[6]. The black box outlines the region encompassing key residues His50 and Glu57 where both cavity propensity is high and solubility is medium-low.

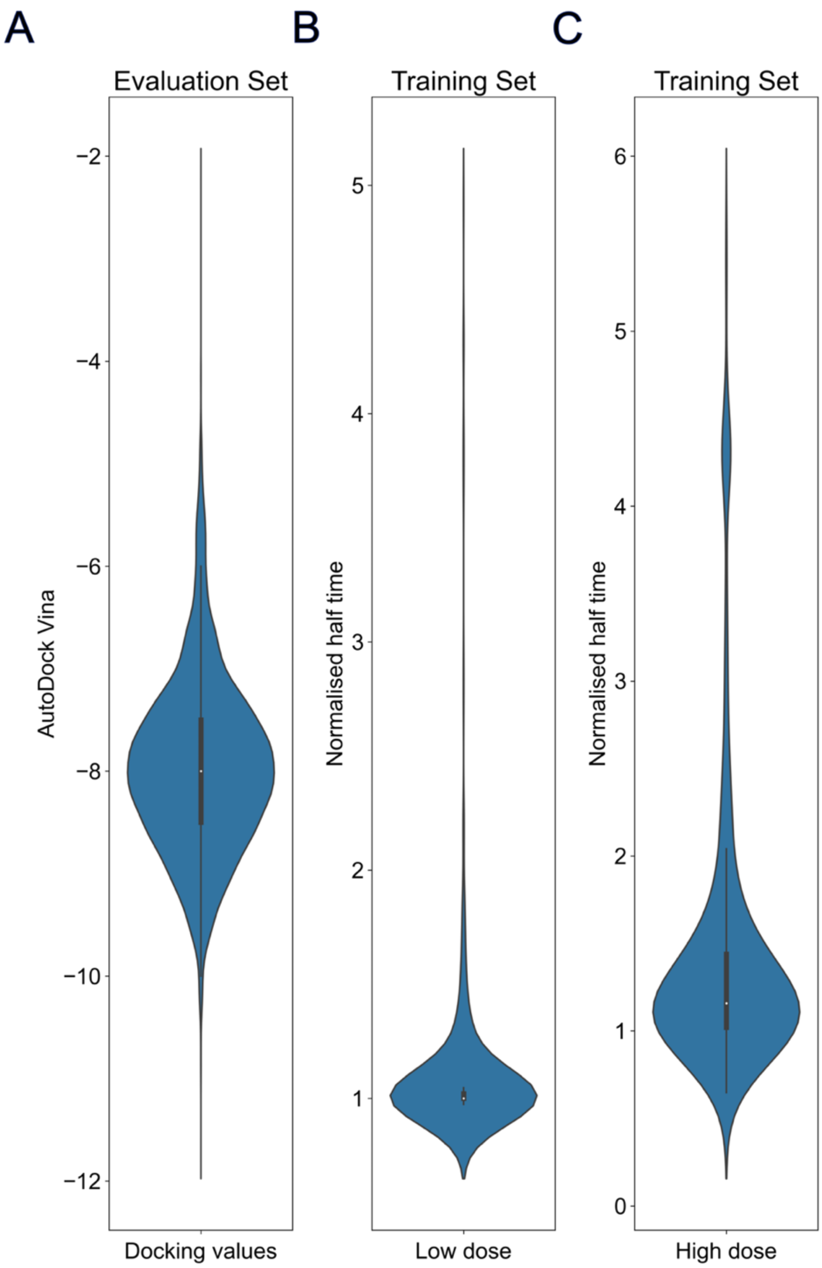

Figure S4. Distributions of the datasets used. (A) AutoDock Vina binding energies (kcal mol^{-1}) for the evaluation set (n=8978 molecules, central measure=median, lower bound=25% of the distribution or Q1, upper bound=75% of the distribution or Q3, lower whisker=Q1-1.5 times interquartile range, upper whisker = Q3+1.5 times interquartile range, furthest points are the lowest and highest values in the distribution). The values are relatively narrowly distributed between -6 and -10 kcal mol^{-1} as the dataset consists of 4 key structures predicted to have good binding. Normalised half times of aggregation at **(B)** 3.12 μM and **(C)** 25 μM for the whole training set (n=447 molecules, all other definitions match **A**), including docking molecules and initial similarity searches and after all iterations had been added. The high dose was used for training in iterations 1-3 and the low dose for iteration 4.

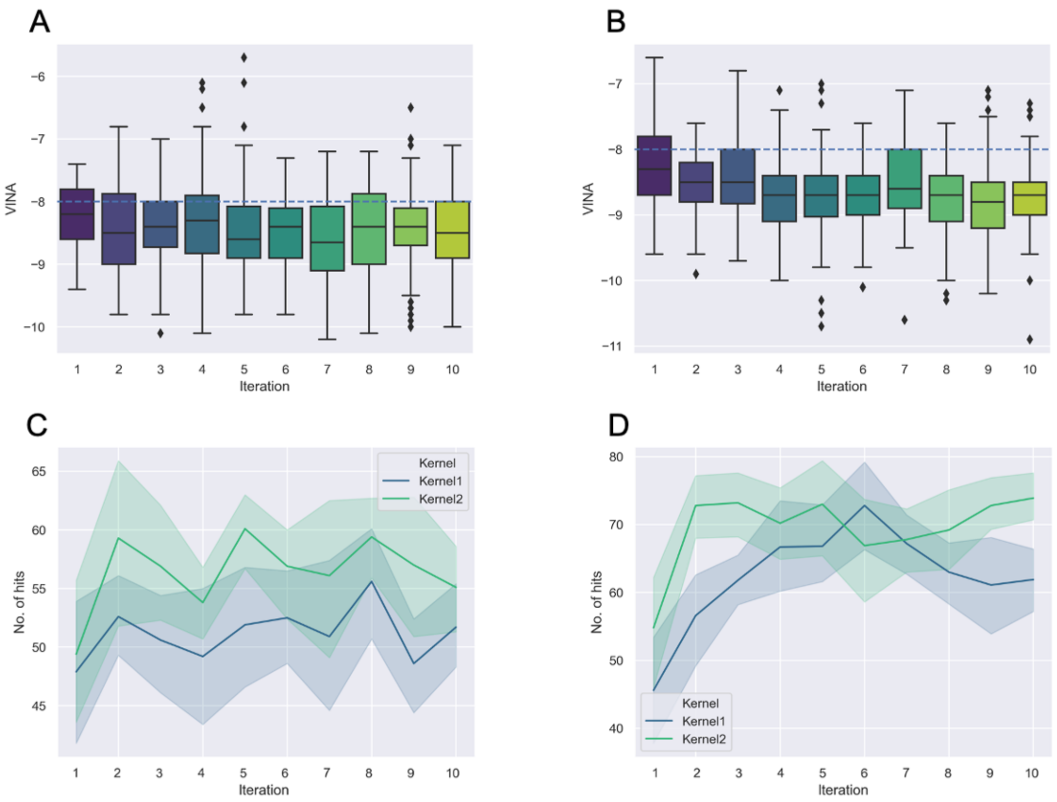

Figure S5. A simulation of the experimental scenario using docking energies as a proxy for aggregation metrics. (A) Starting from a single random sample (n=100 molecules, central measure=median, lower bound=25% of the distribution or Q1, upper bound=75% of the distribution or Q3, lower whisker=Q1-1.5 times interquartile range, upper whisker=Q3+1.5 times interquartile range, points are data that fall outside this range), the GP with RBF kernel was trained and then used to predict the next 100 top molecules from the remaining data (n=8978 molecules). AutoDock Vina binding energies in kcal mol^{-1} are plotted against iteration number, which are shaded from low (purple) to high (yellow). Each boxplot visualises the distribution of binding scores for the top 100 molecules predicted by the algorithm at each iteration. The dotted line indicates the mean binding energy of the evaluation set. **(B)** Same process as in panel **A** with matching definitions and n, but employing the GP with a Matérn Kernel. **(C)** Aggregated average number of hits out of the top 100 predicted molecules (central measure=mean, error=SD) from 10 different random starts of the process shown in panels **A** and **B** for the RBF kernel (Kernel 1, in blue) and the Matérn kernel (Kernel 2, in green). A hit was taken as a molecule falling in the lower quartile of the evaluation set distribution (<-9 kcal/mol). Results were obtained using the half-length representation of the molecules. **(D)** Same process as described in panel **C**, but employing the full-length molecule representation (central measure=mean, error=SD).

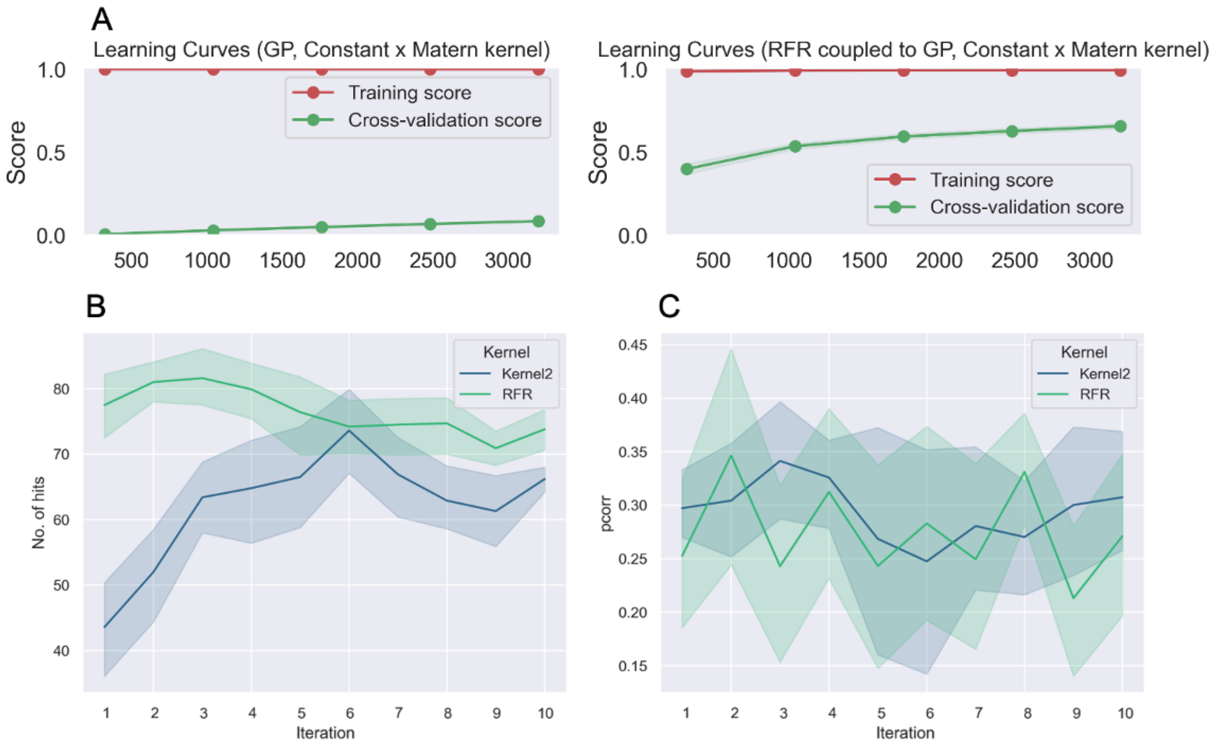

Figure S6. Performance of the RFR method coupled to the Matérn kernel compared to the Matérn kernel alone. (A) R^2 score with increasing training set size (n=up to 4000, central measure=mean, error=SD) for both models, using the full-length representation. On the left is the GP with Matérn kernel alone, and on the right is the GP with Matérn kernel + RFR. Cross validation with 10 random shuffle splits and 20% of the data randomly selected as a validation set. **(B)** Aggregated average hit data from 10 different random starts of the experimental simulation for the iterative approach, starting from 100 randomly selected molecules (central measure=mean, error=SD) and successively adding the actual docking data of the predicted top 100 hits to the training set with each iteration. GP with Matérn kernel alone (Kernel 2 = Matérn, blue) vs GP with Matérn kernel + RFR (green). **(C)** Average Pearson's correlation coefficient (pcorr) between the predicted binding score values and the real scores at each iteration (central measure=mean, error=SD).

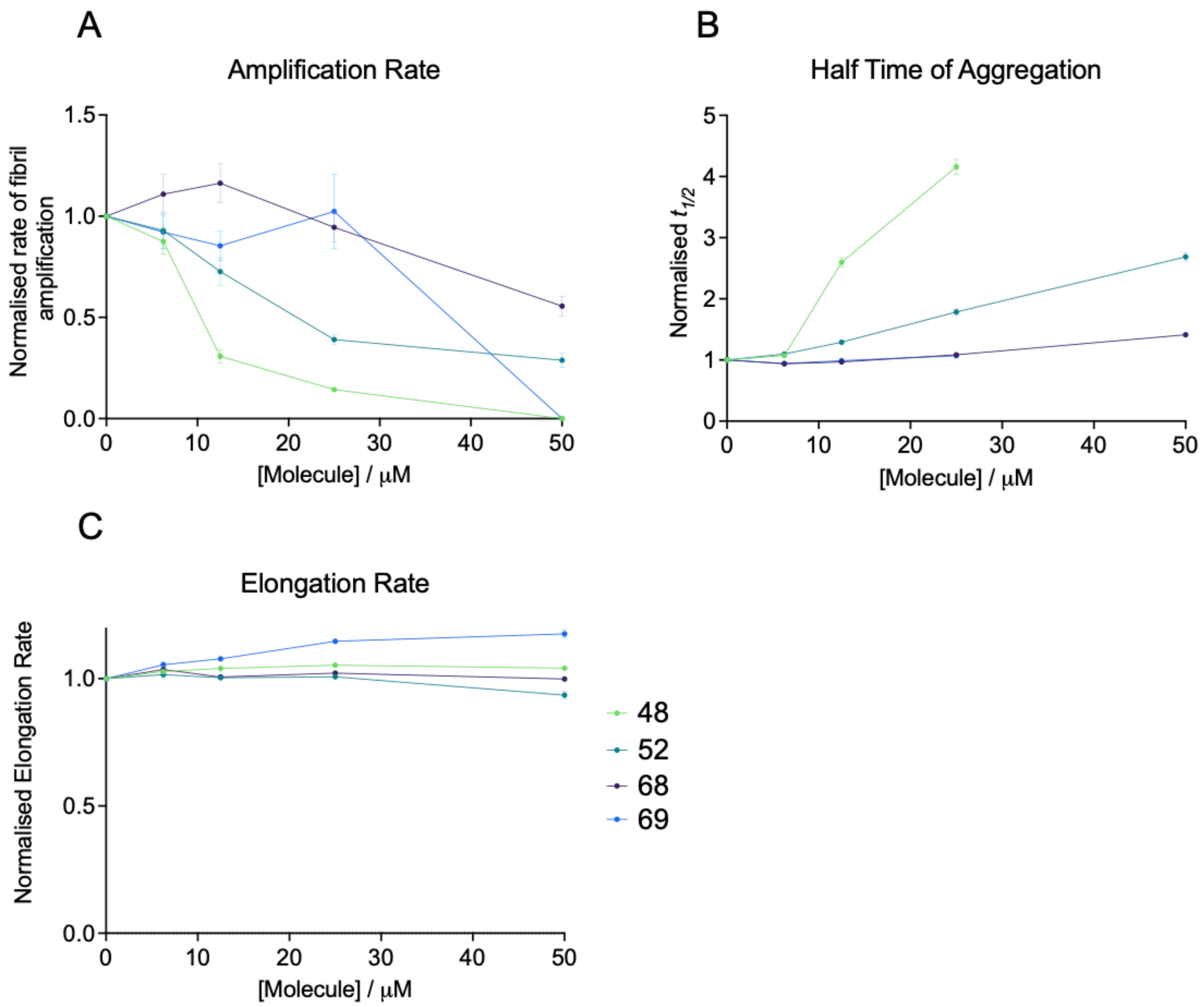

Figure S7. Amplification rate and half time of aggregation of αS in the presence of the 4 molecules in the docking set. (A) Relative rate of fibril amplification of αS in the presence of the 4 docking molecules (labeled as 48, 52, 68 and 69) in the docking set; the kinetic traces are normalised to the DMSO control (n=3 replicates, central measure=mean, error=SEM). **(B)** Half times of aggregation derived from the same experiment (n=3 replicates, central measure=mean, error=SEM). (**C**) Relative rate of fibril elongation normalised to the DMSO control (n=3 replicates, central measure=mean, error=SEM). The amplification rate **A** and half time of aggregation **B** were tested in the machine learning method as parameters to describe the potency of a molecule. The amplification rate tends to be more affected by perturbations to the early slope of the exponential phase that can have large effects on the derived rate value. The half time, although a simpler measure, is more robust and so was chosen for the machine learning approach. Data obtained from reference[1].

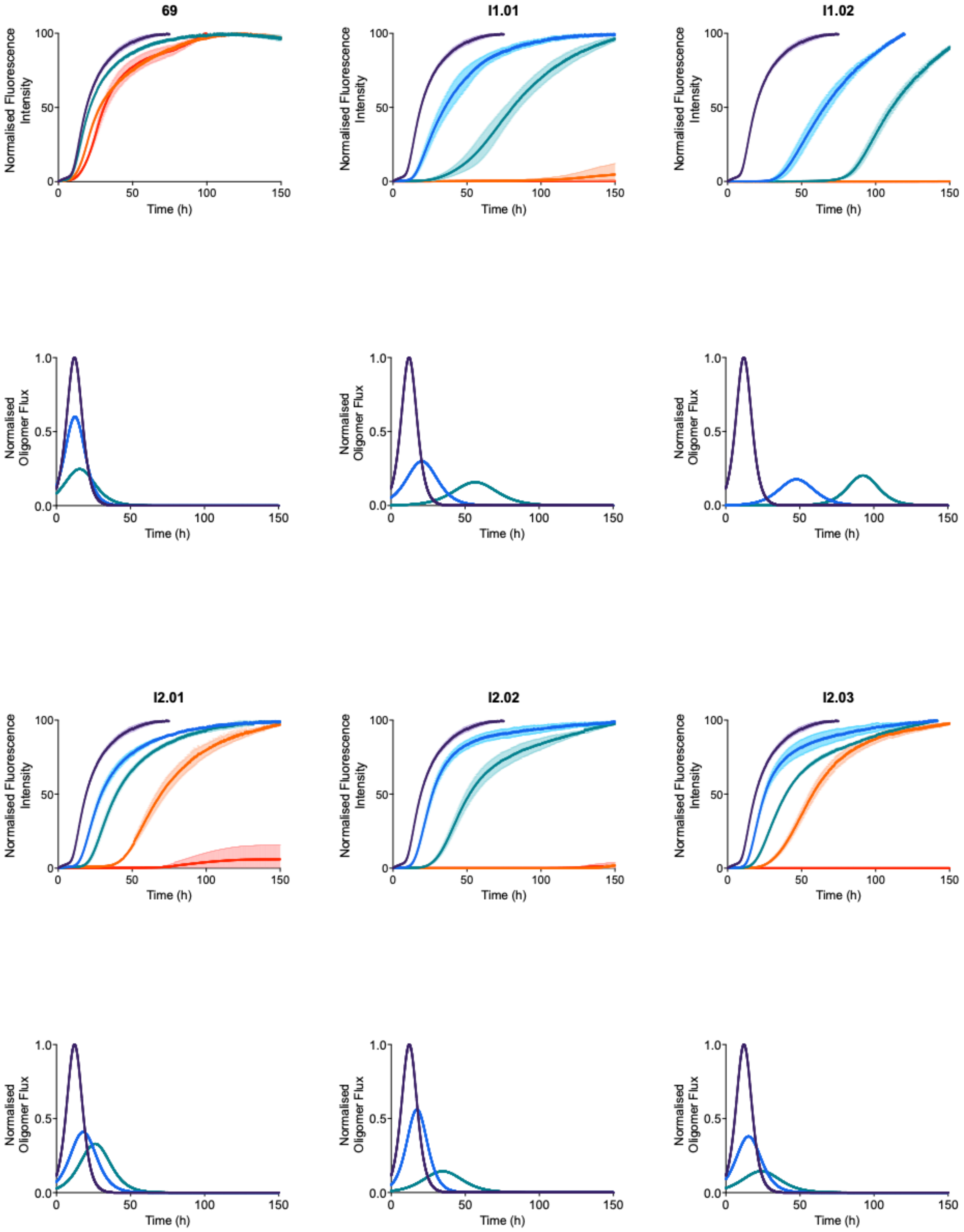
69
I1.01
I1.02
I2.01
I2.02
I2.03
Normalised Fluorescence Intensity
Normalised Oligomer Flux
Time (h)
0
50
100
150
0.0
0.5
1.0

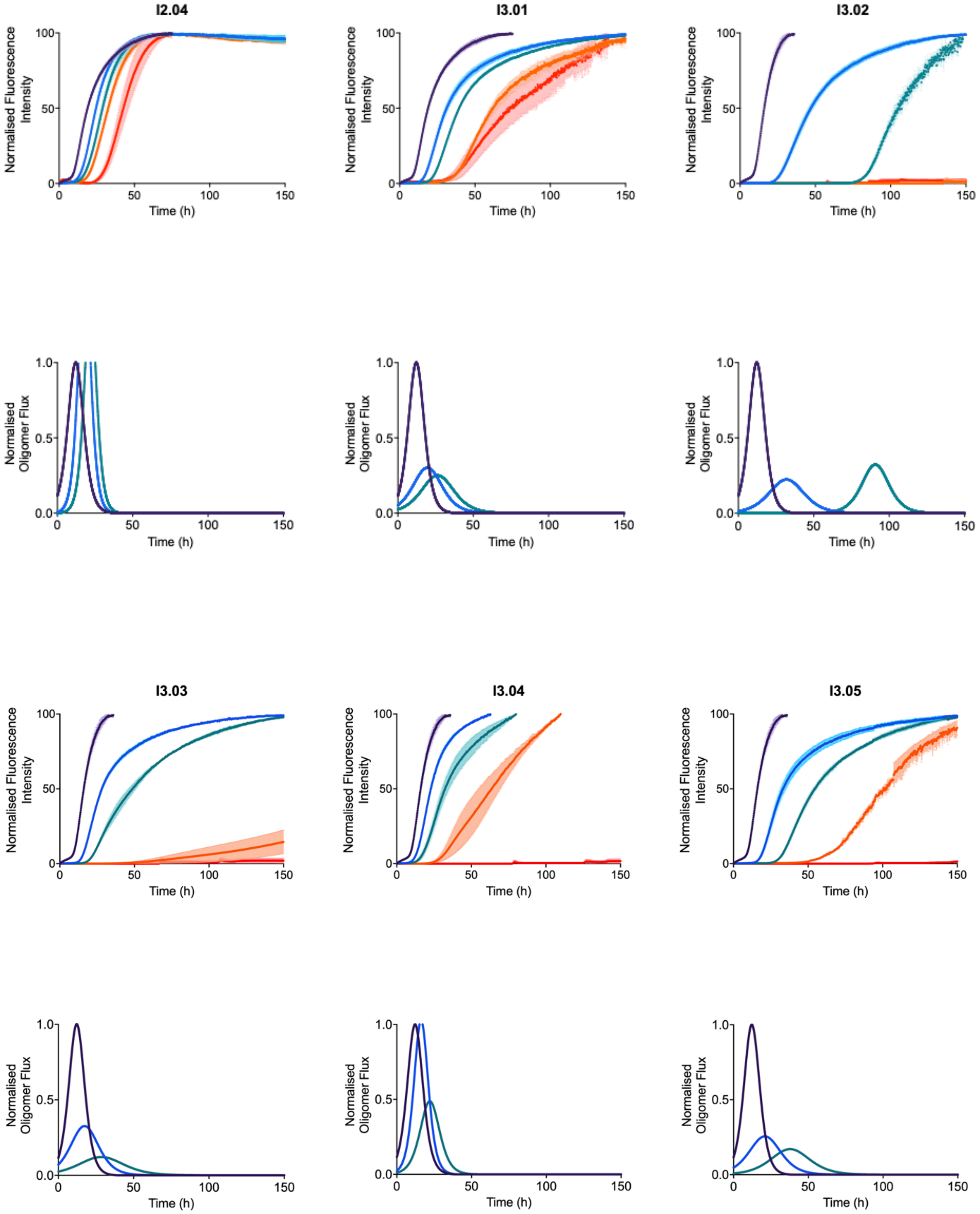
I2.04
I3.01
I3.02
I3.03
I3.04
I3.05
Normalised Fluorescence Intensity
Normalised Oligomer Flux
Time (h)
0
50
100
150
0.0
0.5
1.0

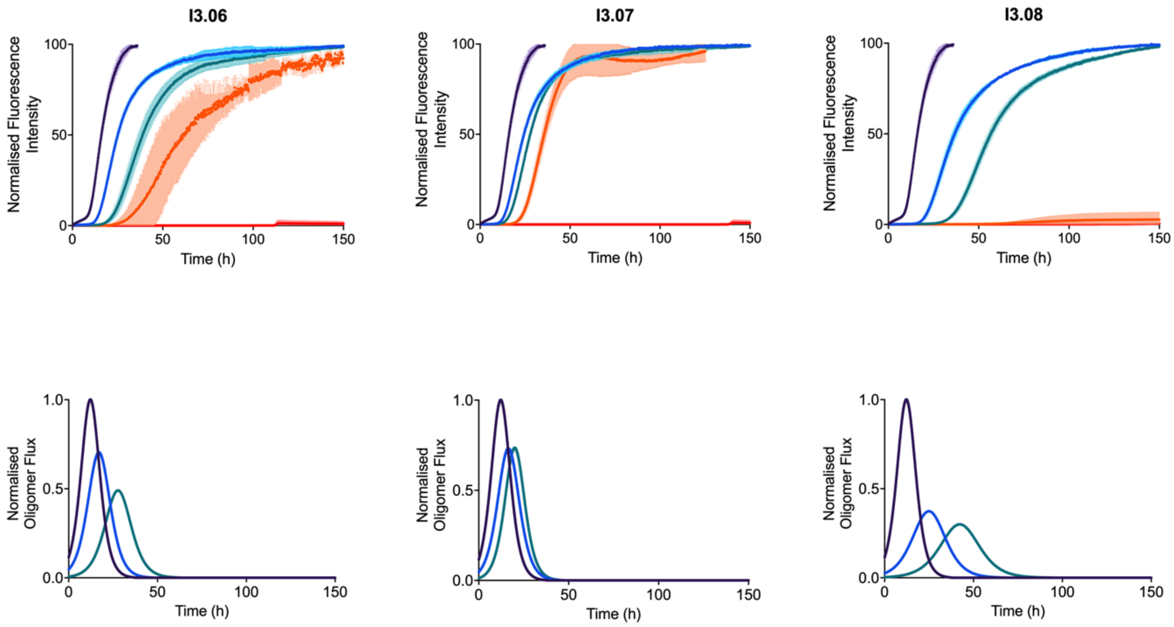

Figure S8. Aggregation curves (top) and oligomer flux simulations (bottom) for the most potent compounds from all of the iterations. The kinetic traces show a 10 μM solution of αS (n=3 replicates, central measure=mean, error=SD) in the presence of 25 nM seeds at pH 4.8, 37 °C in the presence of molecules at 3.12 μM (blue), 6.25 μM (teal), 12.5 μM (orange) and 25 μM (red) versus 1% DMSO alone (dark purple), with endpoints normalised to the αS monomer concentration detected via the Pierce™ BCA Protein Assay at the end of the experiment. Oligomer simulations were carried out only for the lower 2 concentrations, as full aggregation curves were only consistently obtained for all molecules in the secondary nucleation assay at these concentrations.

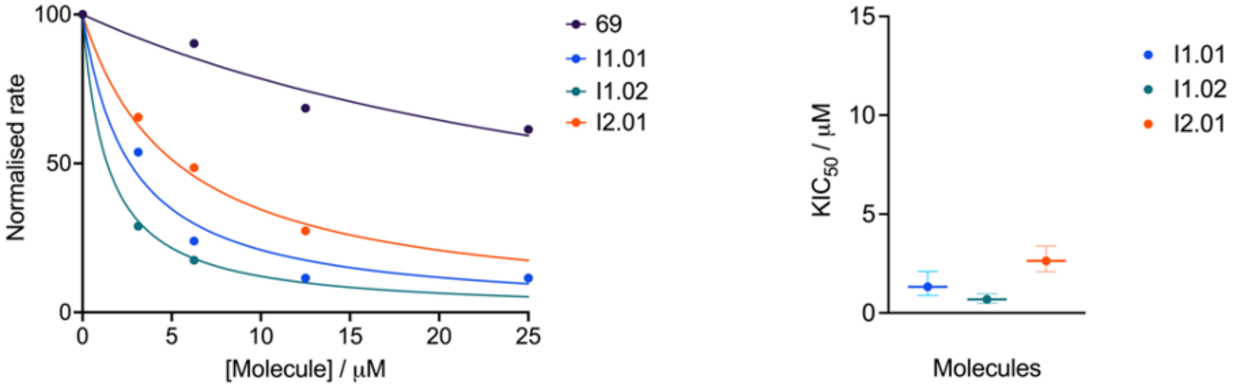
100
50
0
Normalised rate
0 5 10 15 20 25
[Molecule] / µM
69
I1.01
I1.02
I2.01
15
10
5
0
KIC50 / µM
Molecules
I1.01
I1.02
I2.01

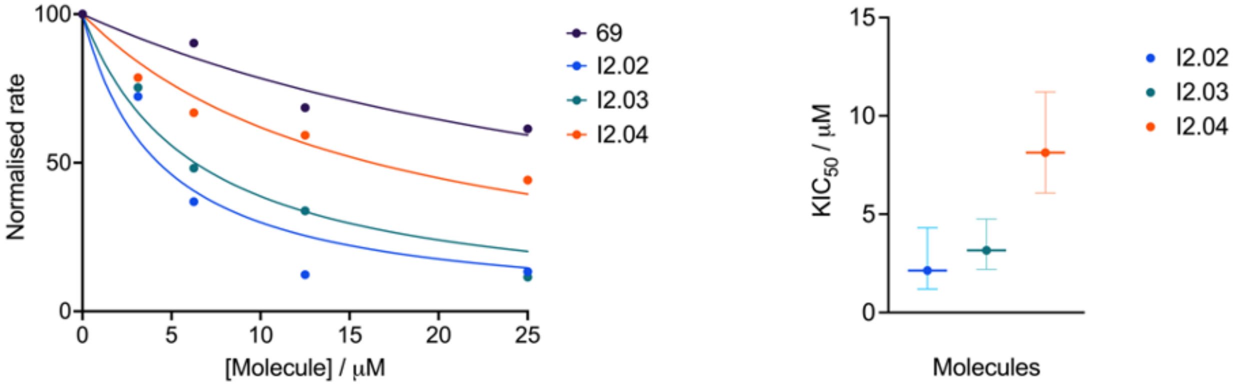
100
50
0
Normalised rate
0 5 10 15 20 25
[Molecule] / µM
69
I2.02
I2.03
I2.04
15
10
5
0
KIC50 / µM
Molecules
I2.02
I2.03
I2.04

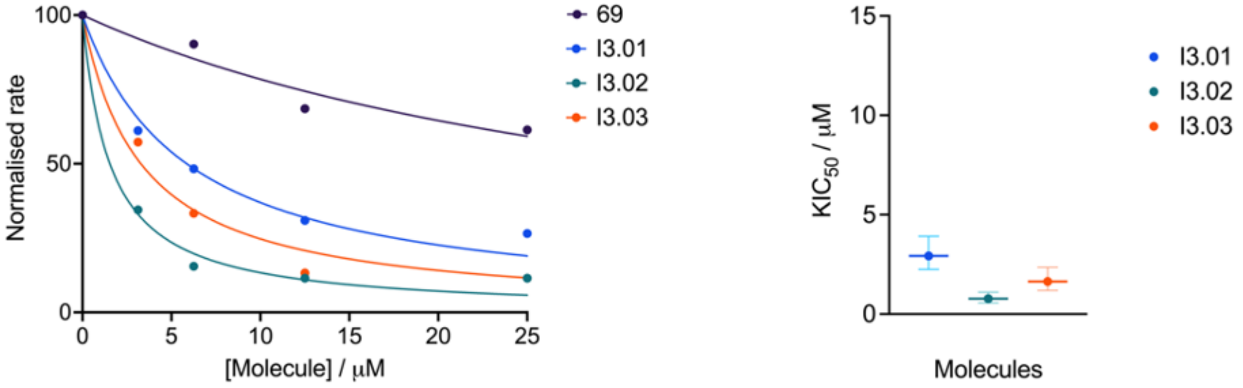
100
50
0
Normalised rate
0 5 10 15 20 25
[Molecule] / µM
69
I3.01
I3.02
I3.03
15
10
5
0
KIC50 / µM
Molecules
I3.01
I3.02
I3.03

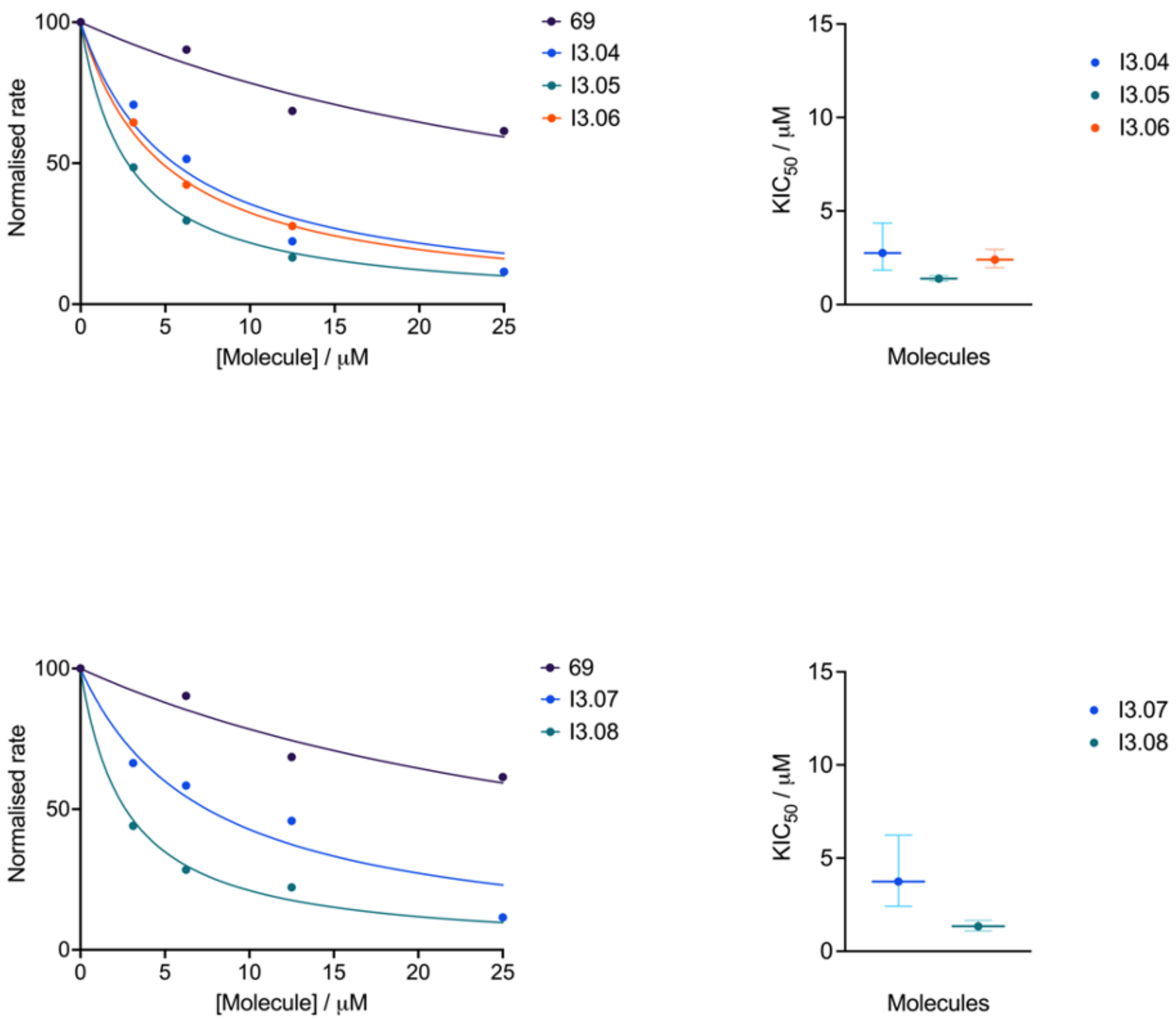

Figure S9. Concentration dependence of the reaction rate (derived from Figure S8) and corresponding 50% kinetic inhibitory concentration (KIC_{50}) values for the most potent compounds (n=3 replicates, central measure=mean, error=95% confidence limits). The approximate normalised rate of reaction (taken as $1/t_{1/2}$) is shown on the left for each molecule at each concentration for which a half time could be obtained. For molecules that completely inhibited the aggregation process on the timescale of the experiment, the $t_{1/2}$ in the presence of the highest concentration of molecule (25 μM) was taken to be the length of the experiment. The approximate rates are fitted using an [Inhibitor] vs. normalized response Hill slope. The KIC_{50} values are shown on the right with the 95% confidence interval.

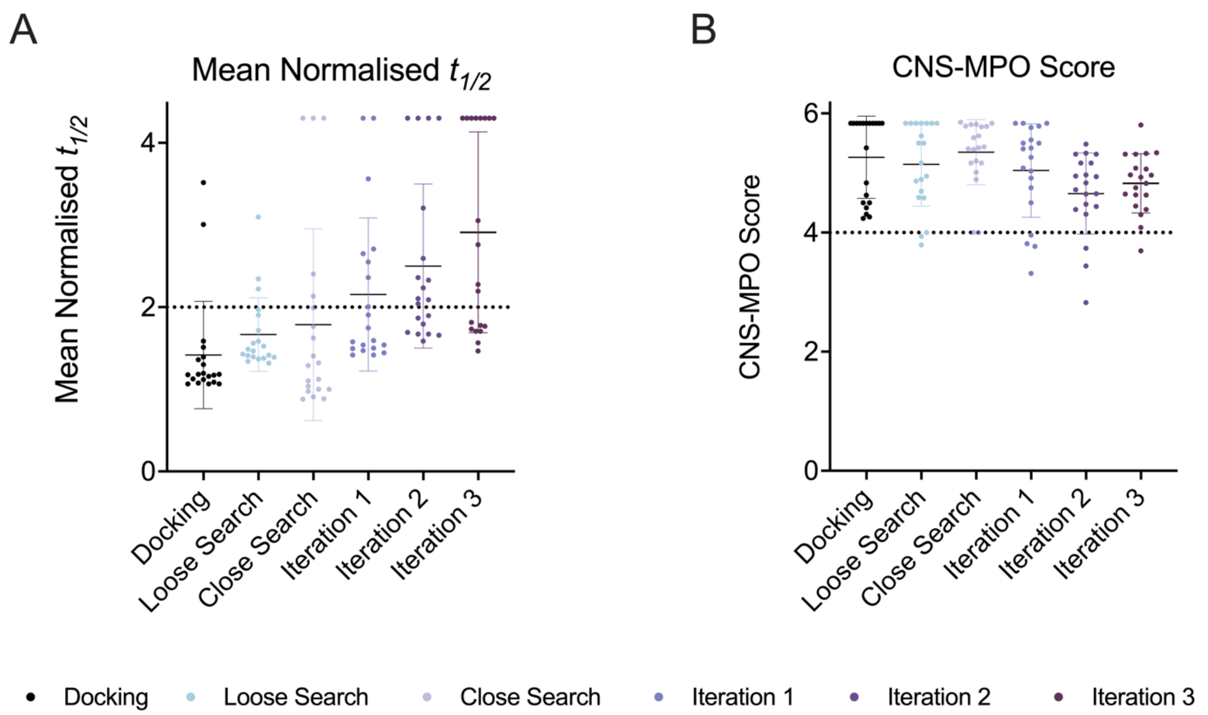

Figure S10. Average $t_{1/2}$ of aggregation and CNS MPO scores for the top 20 molecules at each stage. (A) The stages are the initial docking simulation (68 molecules tested), loose search (69 molecules tested), close search (25 molecules tested), iteration 1 (64 molecules tested), iteration 2 (64 molecules tested) and iteration 3 (56 molecules tested). Molecules were tested at a concentration of 25 μM during screening (n=2 replicates, central measure=mean, error=SD). Molecules that completely prevented aggregation were assigned a $t_{1/2}$ value equal to the length of the experiment. **(B)** Calculated CNS MPO scores for the top 20 structures at each stage (central measure=mean, error=SD). A common cut off for CNS MPO score is 4, as indicated by the horizontal dotted line.

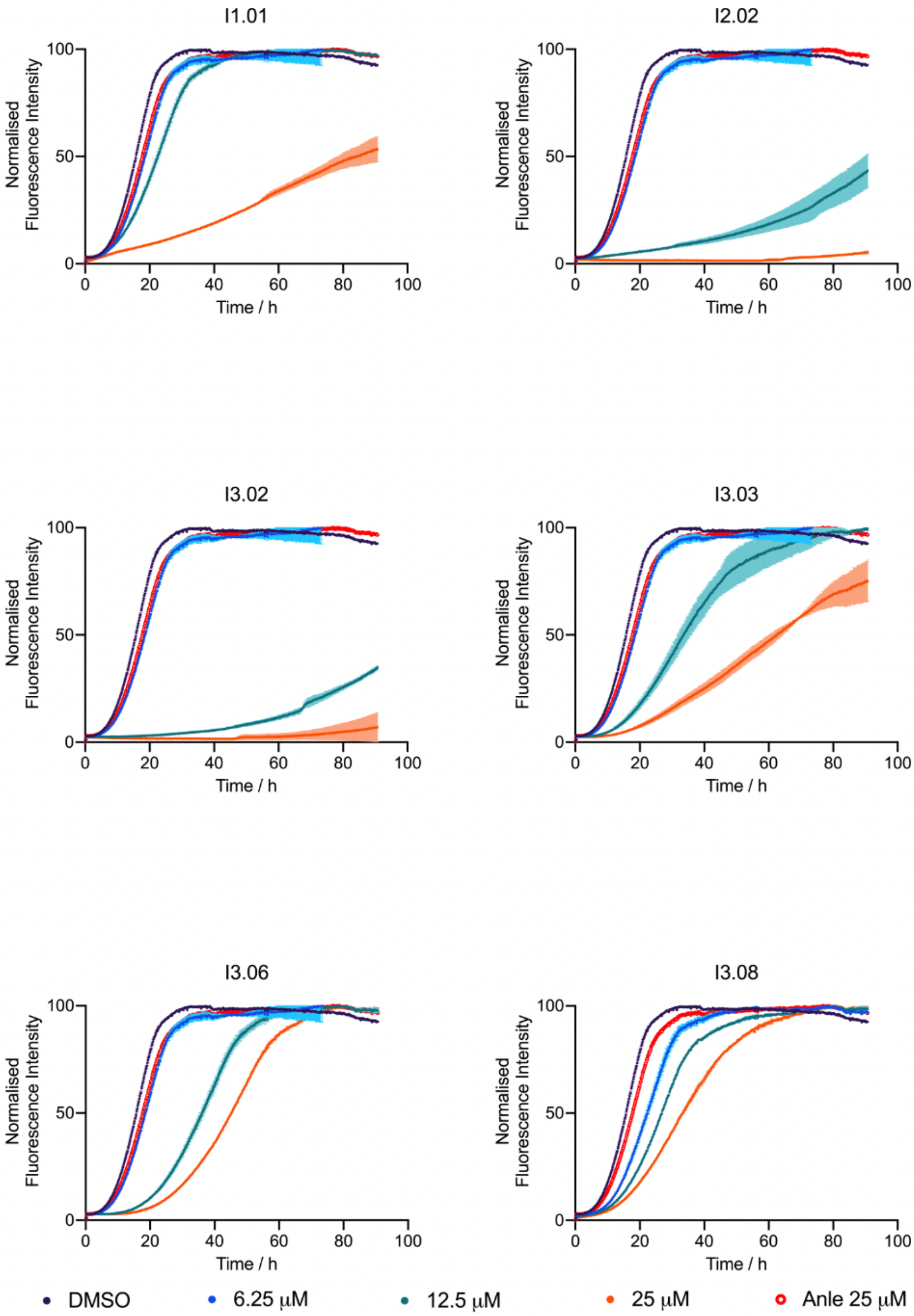

Figure S11. Lipid induced aggregation curves in the presence of the early leads from the project. The kinetic traces show a 20 μM solution (n=3 replicates, central measure=mean, error=SD) of αS plus 100 μM DMPS vesicles (monomer equivalent) at pH 6.5, 30 ^{o}C in the presence of lead molecules at 6.25 μM (blue), 12.5 μM (teal), 25 μM (orange) and Anle-138b at 25 μM (red circles) versus 1% DMSO alone (dark purple), with endpoints normalised to the αS monomer concentration detected via the Pierce™ BCA Protein Assay at the end of the experiment.

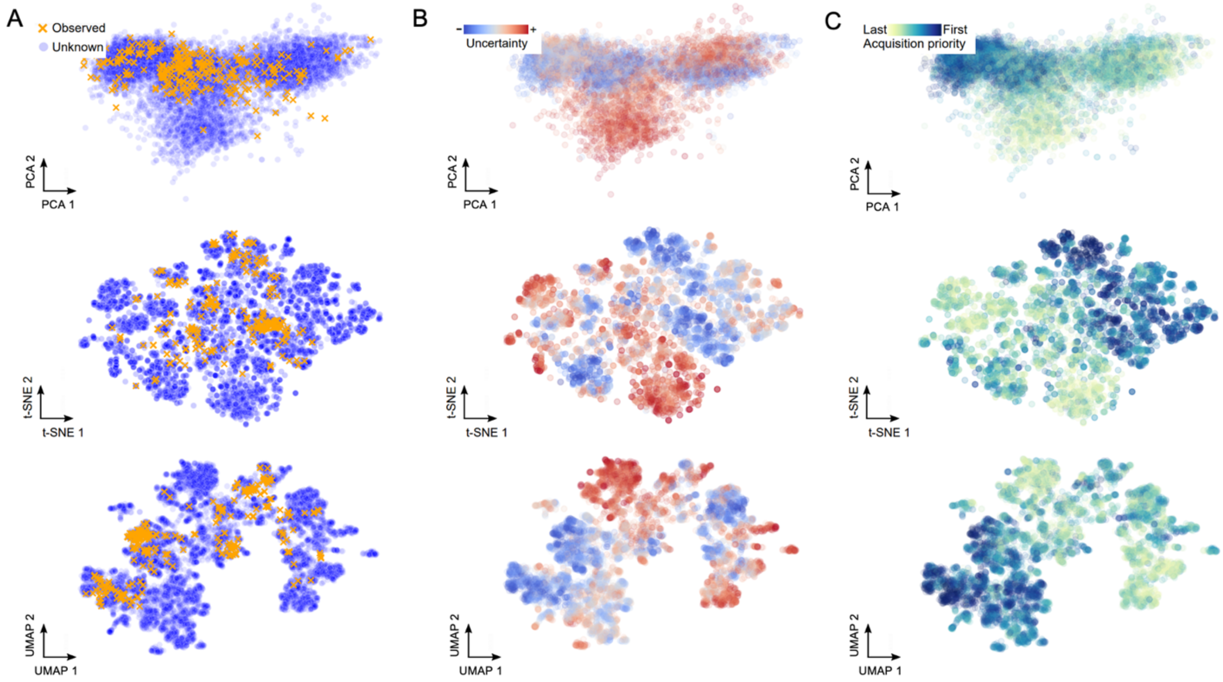

Figure S12. PCA, t-SNE and UMAP visualisations of the compound feature space using uncertainty. (A) From top to bottom: PCA, t-SNE and UMAP visualisations of the compound space indicating which areas of the chemical space have been explored (orange crosses) and which have not (blue circles). **(B)** GPR assigned lower uncertainty (blue) to regions of the chemical space near to the observed data and high uncertainty (red) to areas which were further away. **(C)** The lower uncertainty compounds were prioritised (dark blue) during acquirement ranking.

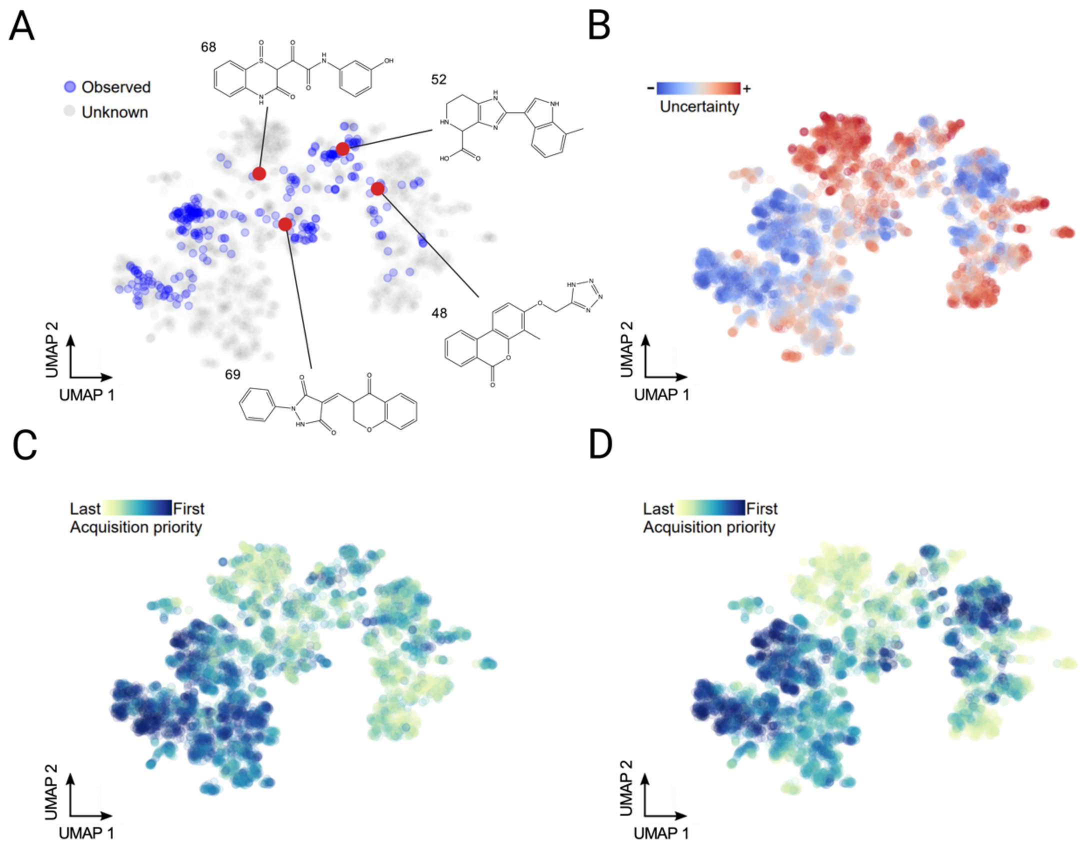

Figure S13. UMAP visualisation of the compound feature space using uncertainty. (A) The visualisation indicates the molecules in the chemical space (grey) that have been tested over the course of the project (blue circles) starting from the 4 initial docking molecules (red circles) in the docking set, and the relative positioning of the parent structures in this space. **(B)** GPR assigned lower uncertainty (blue) to regions of the chemical space near to the observed data and high uncertainty (red) to areas which were further away. **(C)** Acquirement ranking with a low uncertainty penalty. The lower uncertainty compounds were prioritised (dark blue) during acquirement ranking. **(D)** Acquirement ranking with a high uncertainty penalty.

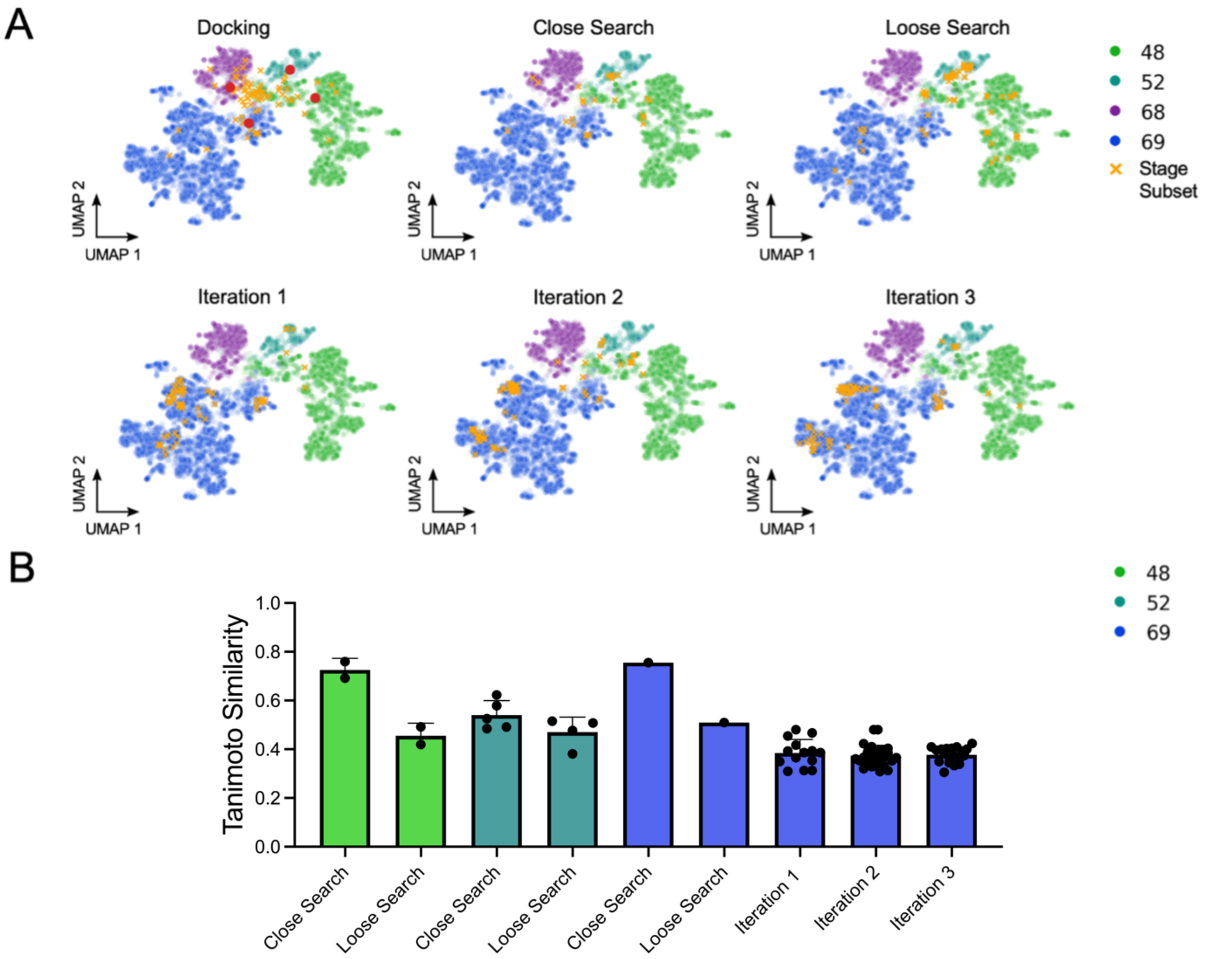

Figure S14. Analysis of the structural changes in the compound optimisation. (A) UMAP visualisation of the compound space indicating how the positioning of each new molecule subset (orange crosses) changed at each stage of the project as well as how the chemical landscape was split between the parent molecules (different colours). The locations of the parent molecules are also indicated in the 'Docking' pane (red circles). **(B)** Average Tanimoto similarity of the more active molecules to their respective parents at each stage of the project (central measure=mean, error=SD). At iterations 1, 2 and 3 all of the leads were derived from molecule 69, albeit with lower similarity than any of the previous stages. Molecule 68 failed to produce any leads outside of the parent molecule.

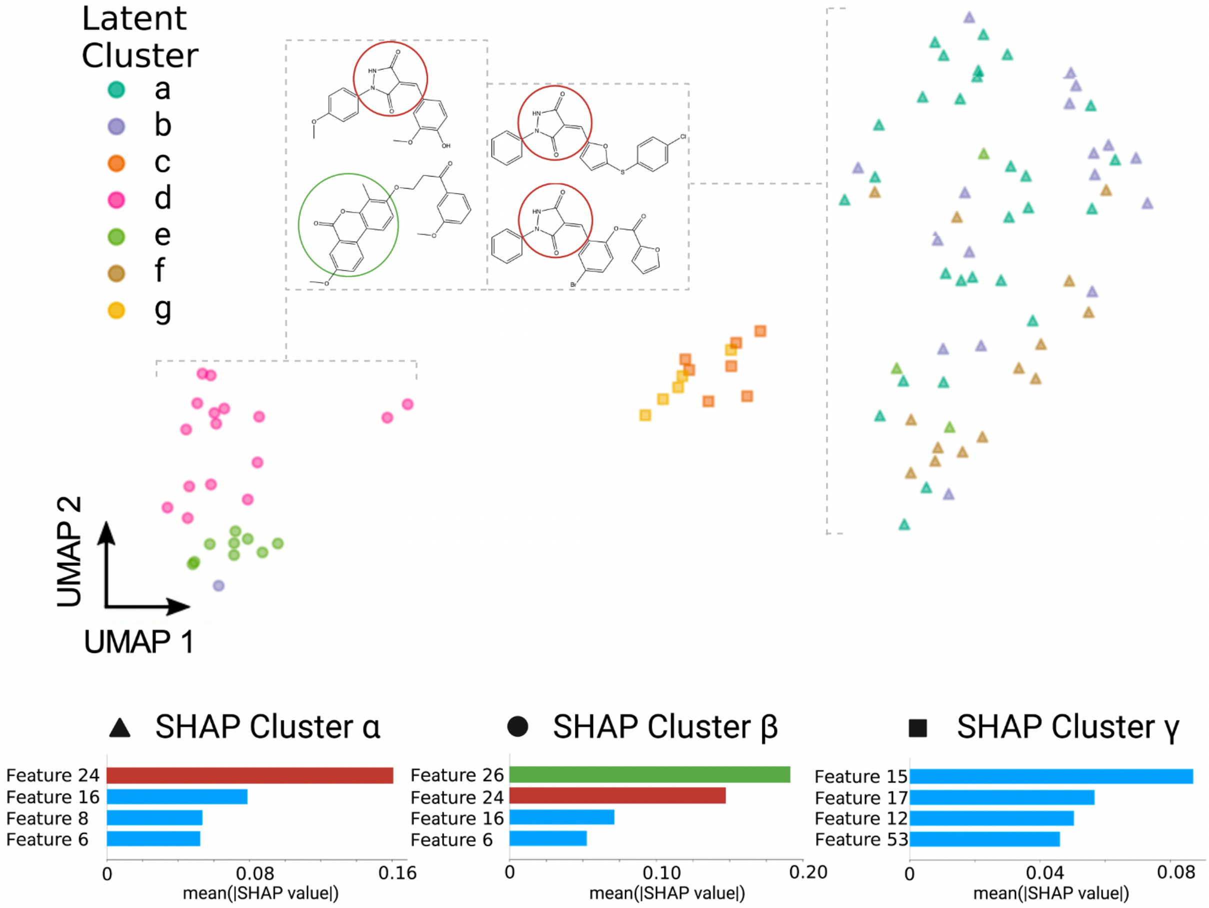

Figure S15. Clustering molecules based on SHAP dimensions and latent vectors. Three SHAP clusters were selected based on clear separation shown by UMAP. The colouring on the UMAP plot is based on the latent space clusters (a-g) and the shape of the marker is based on the SHAP value clustering (α, β, γ). Examining the plot shows that there is no separation between latent clusters c and g, which are grouped together in SHAP cluster γ. Molecules which belonged to latent clusters a, b, and f were mostly grouped together by SHAP clustering, latent cluster e was grouped together with latent cluster d, while latent cluster c was grouped with cluster g. Examination of the top dimensions of each SHAP cluster revealed that dimension 24 at least partly encodes for the key sub-structure of clusters a, b, e and f (3,5-pyrazolidinedione, highlighted in dark red), while dimension 26 at least partly encodes for the key sub-structure of cluster d (the oxygen-rich chromenone fused ring system, highlighted in dark green), and dimensions 15, 17, 12 at least partly encode for the key sub-structure of clusters c and g (carboxylic acid bearing aromatic group).

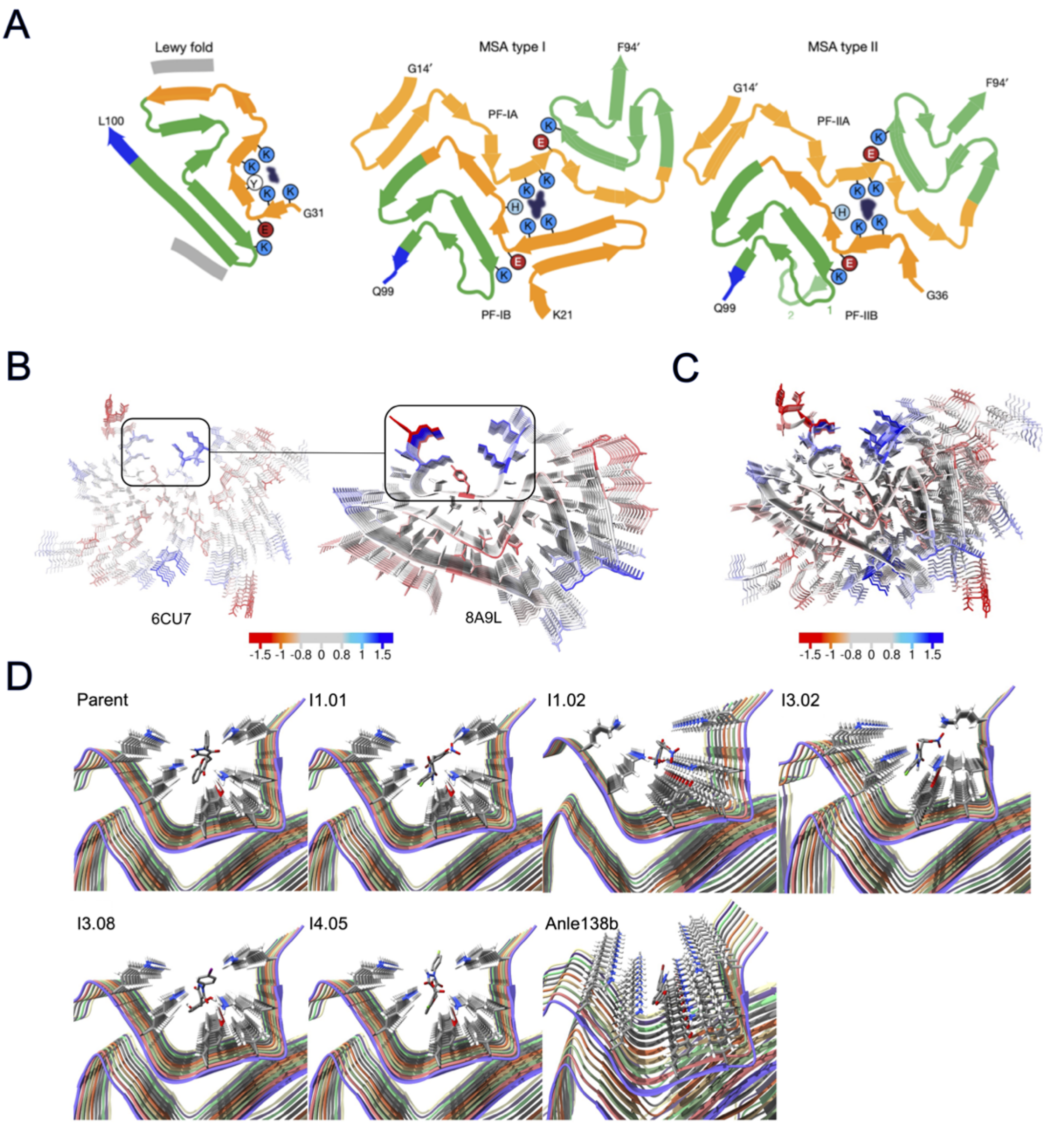

Figure S16. Fibril structures, potential binding pockets and simulated binding poses of I4.05. (A) Folds of the prevalent fibril polymorph in diseased brain material identified via cryo-EM in Parkinson's disease and dementia with Lewy bodies (8A9L), vs multiple system atrophy type I (6XYO) and type II (6XYP) polymorphs. A common motif of 4 lysines enclosing an aromatic side chain (tyrosine in the Lewy fold and histidine in the MSA fold and 6CU7 fold) is observed in the polymorphs, with unidentified electron density in the pocket in each case (adapted from Yang, Y. *et al.*[11]). **(B)** Comparison of the cryo-EM structures of the 6CU7 (recombinant, initially targeted) and 8A9L (brain derived) with the homologous binding site indicated. **(C)** Structural overlap of the 6CU7 and 8A9L fibril structures, with the binding site in 6CU7 aligned with the similar binding site in 8A9L at the top of the diagram. The structures are coloured according to the CamSol residue solubility score[6]. **(D)** Schematics of the molecules bound in their lowest energy state within the 8A9L predicted binding site.

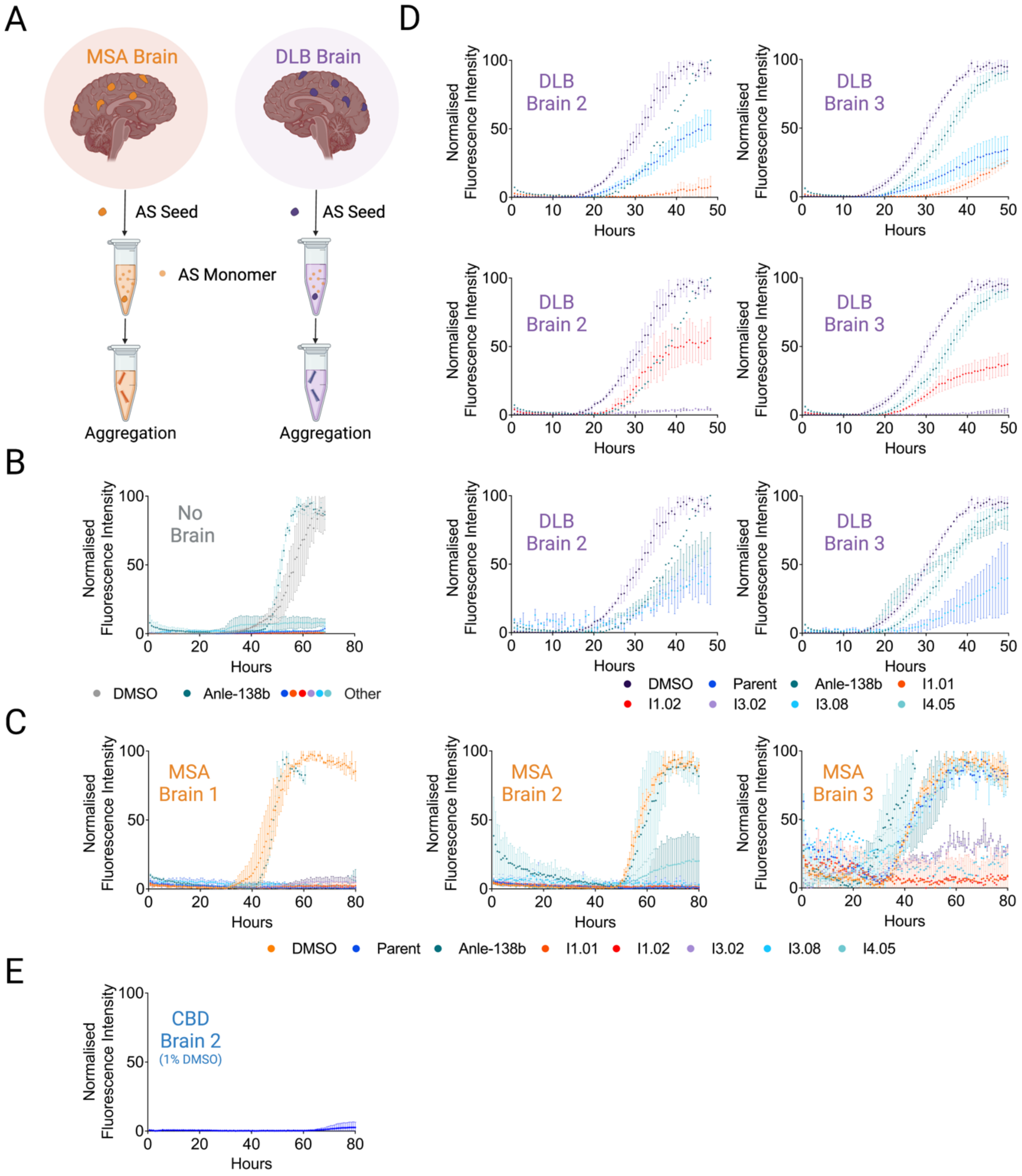

Figure S17. RT-QuIC brain seeding assay. (A) Schematic representation of the RT-QuIC assay, aggregates derived from the brain tissue of patients suffering with multiple system atrophy (MSA) or dementia with Lewy bodies (DLB) were used to induce αS aggregation. **(B)** Kinetic traces of a 7 µM solution of αS in the absence of seeds (pH 8, 42°C, shaking at 400 rpm with 1 min intervals, n=3 replicates, central measure=mean, error=SD). Unseeded samples were 1% DMSO (grey), 7 µM Anle-138b (teal), parent (blue), I1.01 (red), I3.02 (lilac), I3.08 (turquoise) and I4.05 (light blue). Anle-138b, in teal, induced aggregation under this condition. **(C)** Kinetic traces of a 7 µM solution of αS in the presence of MSA seeds (n=3 replicates, all other

conditions match **B**, central measure=mean, error=SD). The MSA samples were 1% DMSO (light orange), 7 μM Anle-138b (teal), parent (blue), I1.01 (red), I3.02 (lilac), I3.08 (turquoise) and I4.05 (light blue). Anle-138b had no effect in samples 1 and 2 but appeared to accelerate aggregation in sample 3. **(D)** Kinetic traces of a 7 μM solution of αS in the presence of DLB seeds (n=3 replicates, all other conditions match **B**, central measure=mean, error=SD). The DLB samples were 1% DMSO (purple), 7 μM Anle-138b (teal), parent (blue), I1.01 (red), I3.02 (lilac), I3.08 (turquoise) and I4.05 (light blue). Data have been separated for clarity. The DMSO and Anle-138b traces are shown on each graph, with 2 molecules from the docking or ML shown for comparison: Parent and I1.01 (top), I1.02 and I3.02 (middle), I3.08 and I4.05 (bottom). Anle-138b exerts a consistent mild inhibition for these two brain samples. **(E)** Kinetic traces of a 7 μM solution of αS in the presence of CBD seeds (n=3 replicates, all other conditions match **B**, central measure=mean, error=SD) and 1% DMSO over a longer time course. No significant aggregation was observed over 80 h.

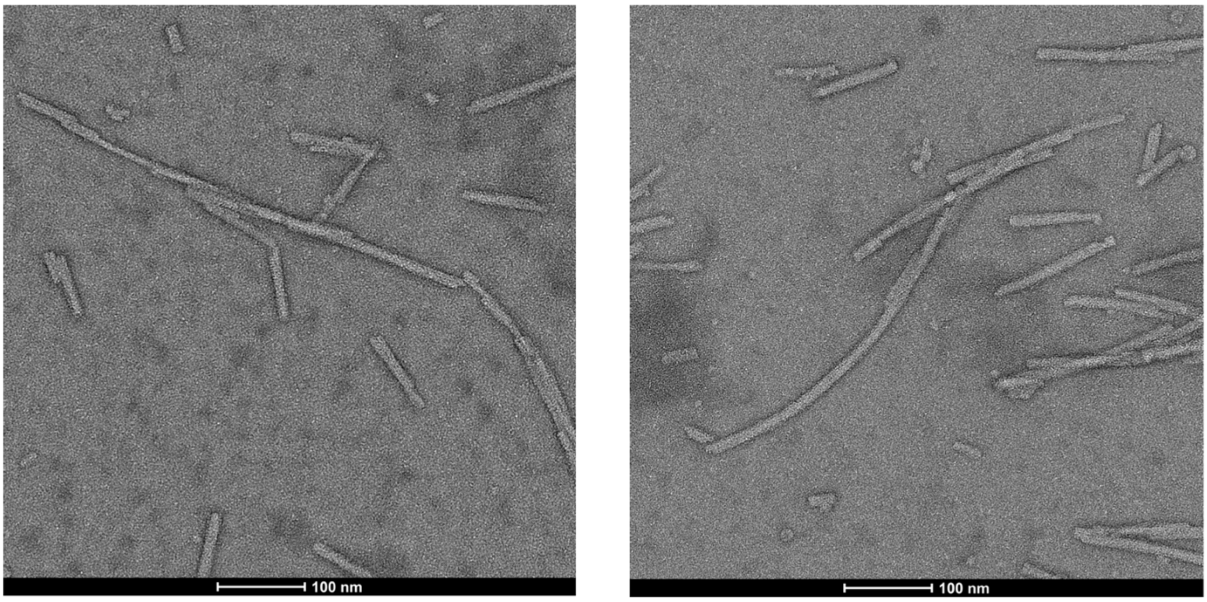

Figure S18. Transmission electron microscopy images of the fibrils at the end of the secondary nucleation assay. Two representative images are shown, the scale bar is 100 nm.

Supplementary References

1. Chia, S. et al. Structure-Based Discovery of Small-Molecule Inhibitors of the Autocatalytic Proliferation of alpha-Synuclein Aggregates. *Mol Pharm* (2022).
2. Trott, O. & Olson, A.J. AutoDock Vina: improving the speed and accuracy of docking with a new scoring function, efficient optimization, and multithreading. *J Comput Chem* **31**, 455-61 (2010).
3. McGann, M. FRED pose prediction and virtual screening accuracy. *Journal of chemical information and modeling* **51**, 578-596 (2011).
4. Li, B. et al. Cryo-EM of full-length α-synuclein reveals fibril polymorphs with a common structural kernel. *Nature communications* **9**, 1-10 (2018).
5. Le Guilloux, V., Schmidtke, P. & Tuffery, P. Fpocket: an open source platform for ligand pocket detection. *BMC Bioinformatics* **10**, 168 (2009).
6. Sormanni, P., Aprile, F.A. & Vendruscolo, M. The CamSol method of rational design of protein mutants with enhanced solubility. *Journal of molecular biology* **427**, 478-490 (2015).
7. McGann, M. FRED and HYBRID docking performance on standardized datasets. *Journal of computer-aided molecular design* **26**, 897-906 (2012).
8. Wang, Z. et al. Comprehensive evaluation of ten docking programs on a diverse set of protein–ligand complexes: the prediction accuracy of sampling power and scoring power. *Physical Chemistry Chemical Physics* **18**, 12964-12975 (2016).
9. Hie, B., Bryson, B.D. & Berger, B. Leveraging Uncertainty in Machine Learning Accelerates Biological Discovery and Design. *Cell Syst* **11**, 461-477 e9 (2020).
10. Robinson, C. & Dilkina, B. A machine learning approach to modeling human migration. in *Proceedings of the 1st ACM SIGCAS Conference on Computing and Sustainable Societies* 1-8 (2018).
11. Yang, Y. et al. Structures of α-synuclein filaments from human brains with Lewy pathology. *Nature* **610**, 791-795 (2022).
