# Supplementary material for: Discovery of potent inhibitors of α-synuclein aggregation using structure-based iterative learning
Source: Nat Chem Biol. 2024 Apr 17;20(5):634–45. doi: 10.1038/s41589-024-01580-x (PMC11062903; doi:10.1038/s41589-024-01580-x)
Supplement: Supplementary file 2 — Reporting Summary [file 41589_2024_1580_MOESM2_ESM.pdf]

nature portfolio

Corresponding author(s): Michele Vendruscolo

Last updated by author(s): Oct 28, 2023

Reporting Summary

Nature Portfolio wishes to improve the reproducibility of the work that we publish. This form provides structure for consistency and transparency in reporting. For further information on Nature Portfolio policies, see our Editorial Policies and the Editorial Policy Checklist.

Statistics

For all statistical analyses, confirm that the following items are present in the figure legend, table legend, main text, or Methods section.

n/a	Confirmed	
☐	☒	The exact sample size (*n*) for each experimental group/condition, given as a discrete number and unit of measurement
☐	☒	A statement on whether measurements were taken from distinct samples or whether the same sample was measured repeatedly
☒	☐	The statistical test(s) used AND whether they are one- or two-sided<br>*Only common tests should be described solely by name; describe more complex techniques in the Methods section.*
☐	☒	A description of all covariates tested
☐	☒	A description of any assumptions or corrections, such as tests of normality and adjustment for multiple comparisons
☐	☒	A full description of the statistical parameters including central tendency (e.g. means) or other basic estimates (e.g. regression coefficient) AND variation (e.g. standard deviation) or associated estimates of uncertainty (e.g. confidence intervals)
☒	☐	For null hypothesis testing, the test statistic (e.g. *F*, *t*, *r*) with confidence intervals, effect sizes, degrees of freedom and *P* value noted<br>*Give P values as exact values whenever suitable.*
☐	☒	For Bayesian analysis, information on the choice of priors and Markov chain Monte Carlo settings
☒	☐	For hierarchical and complex designs, identification of the appropriate level for tests and full reporting of outcomes
☒	☐	Estimates of effect sizes (e.g. Cohen's *d*, Pearson's *r*), indicating how they were calculated

Our web collection on statistics for biologists contains articles on many of the points above.

Software and code

Policy information about availability of computer code

Data collection

RDKit (2020.09.1) was used to carry out similarity searches.

Data analysis

Code location: https://github.com/rohorne07/Iterate
The following software was used, implemented on Python (3.9.7 |Anaconda):
MolVS==0.1.1
MulticoreTSNE==0.1
fbpca==1.0
matplotlib==3.3.4
numpy==1.20.1
pandas==1.2.4
scikit-learn==0.24.1
scipy==1.6.2
seaborn==0.11.1
umap-learn==0.3.10

Note: Python 3.6.13 required for jtnnencoder functioning
torch==1.10.0
rdkit==2020.09.1
scipy==1.5.2

For other analysis and graphing:

GraphPad Prism==9.1.2

For microfluidic device design:
AutoCAD - Autodesk==24.3

For manuscripts utilizing custom algorithms or software that are central to the research but not yet described in published literature, software must be made available to editors and reviewers. We strongly encourage code deposition in a community repository (e.g. GitHub). See the Nature Portfolio guidelines for submitting code & software for further information.

Data

Policy information about availability of data

All manuscripts must include a data availability statement. This statement should provide the following information, where applicable:

- Accession codes, unique identifiers, or web links for publicly available datasets
- A description of any restrictions on data availability
- For clinical datasets or third party data, please ensure that the statement adheres to our policy

The ZINC 15 database (https://zinc15.docking.org/) was used for initial similarity searches around the starting molecules which were carried out using RDKit on Python, using MolVS to standardise SMILES (see versions above). The datasets produced and all of the code are publicly available in a GitHub repository (https://github.com/rohorne07/Iterate).

Human research participants

Policy information about studies involving human research participants and Sex and Gender in Research.

Reporting on sex and gender	Full details are in supplementary table S2. The sex of the patients contributing samples were as follows: DLB (3 males), CBD (1 female) and MSA (2 females, 1 male)
Population characteristics	Full details are in supplementary table S2. Each patient sample is listed here with the sex (M/F), age at death (years), disease duration (years), postmortem interval (hours),primary diagnosis, and additional diagnosis in this order in brackets: DLB sample 1 (M, 81, N.A., 20, diffuse Lewy body disease, senile changes plus cerebrovascular disease), DLB sample 2 (M, 70, 6, N.A., Lewy body Dementia, senile changes plus cerebrovascular disease), DLB sample 3 (M, 75, 4, N.A., Lewy body Dementia, senile changes plus cerebrovascular disease), MSA sample 1 (F, 62, N.A., N.A., MSA, N.A.), MSA sample 2 (F, 71, N.A., N.A., MSA, senile changes plus cerebrovascular disease), MSA sample 3 (M, 52, N.A., 3, MSA, senile changes plus cerebrovascular disease), CBD sample (F, 51, 10, 9.6, CBD, N.A.).
Recruitment	Not applicable, brain samples are provided from their respective sources purely through availability due to the scarcity of these samples.
Ethics oversight	Deidentified post-mortem brain samples were obtained from sources indicated in Table S2. As samples were obtained from deceased, de-identified, consenting individuals, no further ethical approval was required.

Note that full information on the approval of the study protocol must also be provided in the manuscript.

Field-specific reporting

Please select the one below that is the best fit for your research. If you are not sure, read the appropriate sections before making your selection.

☒ Life sciences ☐ Behavioural & social sciences ☐ Ecological, evolutionary & environmental sciences

For a reference copy of the document with all sections, see nature.com/documents/nr-reporting-summary-flat.pdf

Life sciences study design

All studies must disclose on these points even when the disclosure is negative.

Sample size	The maximum number of molecules within the ZINC 15 database with Tanimoto coefficient > 0.3 to the starting hit molecules were used as the molecule library (~8000 molecules). ~160 molecules from the initial docking simulation work and similarity searches comprised the initial training set.
Data exclusions	No exclusions.
Replication	All attempts at replication were successful. All algorithmic work relying on initial random states was repeated from multiple different random states, and for experimental testing molecules were screened in duplicate or triplicate at 25 uM during iterative cycles and strong hits (norm. half time > 4) were re-screened in triplicate at a concentration gradient moving down from 12.5 uM. Another screen was carried out for hits (norm. half time > 2) at a lower concentration (3.12 uM) to further validate compound potency. SPR experiments were arranged in duplicate in a pyramidal concentration arrangement of the small molecule, and repeated at 2 different pHs yielding similar results. RT-QuIC assays were tested in quadruplicate and repeated for 3 different brain samples for each condition. All other experiments were successfully duplicated

	apart from the uFFE which could only be carried out once due to the difficulty and length of the experiment.
Randomization	Data for computational model training/testing were randomly split into training and testing sets. The standard method of 5 k fold splitting was applied. No other efforts at randomization were necessary in this work, the only other area where a variable population was sampled being the individuals to obtain brain samples from, where the availability of samples was too low for randomization to be effective.
Blinding	It was not possible to blind this study as analytical and experimental work was carried out by the same individual, which required knowledge of the chemical matter involved. However, molecule purchase orders were purely based on the algorithm output and the algorithm settings were not altered in response to the results during the process.

Reporting for specific materials, systems and methods

We require information from authors about some types of materials, experimental systems and methods used in many studies. Here, indicate whether each material, system or method listed is relevant to your study. If you are not sure if a list item applies to your research, read the appropriate section before selecting a response.

Materials & experimental systems

n/a	Involved in the study	
☒	☐	Antibodies
☒	☐	Eukaryotic cell lines
☒	☐	Palaeontology and archaeology
☒	☐	Animals and other organisms
☒	☐	Clinical data
☒	☐	Dual use research of concern

Methods

n/a	Involved in the study	
☒	☐	ChIP-seq
☒	☐	Flow cytometry
☒	☐	MRI-based neuroimaging
